# Progress on Silica Pervaporation Membranes in Solvent Dehydration and Solvent Recovery Processes

**DOI:** 10.3390/ma13153354

**Published:** 2020-07-28

**Authors:** Aakash Rajawat, Sundarrajan Subramanian, Seeram Ramakrishna

**Affiliations:** 1 Department of Mechanical Engineering, Birla Institute of Technology and Science, Pilani, K. K. Birla Goa Campus, Zuarinagar, Sancoale, Goa 403726, India; e0511000@u.nus.edu; 2 Center for Nanofibers and Nanotechnology Lab, Mechanical Engineering, National University of Singapore, Blk E3 05-12, 2 Engineering Drive 3, Singapore 117581, Singapore

**Keywords:** silica membrane, pervaporation, inorganic, solvent recovery, solvent dehydration

## Abstract

Separation processes aimed at recovering the solvent from effluent streams offer a means for establishing a circular economy. Conventional technologies such as distillation are energy-intensive, inefficient and suffer from high operating and maintenance costs. Pervaporation based membrane separation overcomes these challenges and in conjunction with the utilization of inorganic membranes derived from non-toxic, sufficiently abundant and hence expendable, silica, allows for high operating temperatures and enhanced chemical and structural integrity. Membrane-based separation is predicted to dominate the industry in the coming decades, as the process is being understood at a deeper level, leading to the fabrication of tailored membranes for niche applications. The current review aims to compile and present the extensive and often dispersive scientific investigations to the reader and highlight the current scenario as well as the limitations suffered by this mature field. In addition, viable alternative to the conventional methodologies, as well as other rival materials in existence to achieve membrane-based pervaporation are highlighted.

## 1. Introduction

Solvents are very critical not only to produce lifesaving drugs, but also to manufacture many household products. Hence, they are widely used in the pharmaceutical industry, petroleum refineries, paints and coatings, adhesives, printing inks, industrial cleaning and research essential to the fields of medicine and disease diagnosis. The scale at which the solvents are utilized to realize mass-manufactured products leads to difficulties and inefficiencies in their handling and disposal, essentially, when a large section of these are toxic to the humans as well as their potential to corrode thriving ecosystems by their unchecked penetration into the environment.

Recycling of the constituents comprising of the waste products can help to reduce the risk of environmental degradation and will bring down the costs associated with the raw materials. This approach makes the whole manufacturing process more eco-friendly and economical. Several conventional technologies such as distillation, pressure swing distillation and fractional distillation exist in place to deal with the processing of the solvent effluent wastes and hence minimize their impact on the environment. The oldest and most exploited technique is fractional distillation. Although its wide utilization across the industry has been largely unchallenged, it is plagued by its non-viability for azeotrope differentiation, dilute aqueous organic solutions, thermally degradable organic compounds [1], requirement of large separation energy (reflux ratio + 1) × (heat of evaporation) [2,3] and less efficiency due to high irreversibility. Recently, modern separation techniques have been extensively researched and are beginning to gain acceptance towards their viability as the next separation methodology for large industrial usage.

Pervaporation separation or dehydration of aqueous organic mixtures though membranes has proved to be the most reliable and efficient method. The limitations of distillation are surpassed by this method due to its ability to separate azeotrope mixtures, thermally degradable mixtures, stable performance against dilute aqueous organic mixtures, requirement of low device volume and energy consumption as well as simple process setup and potential for continuous operation. Pervaporation has an enormous potential for revolutionizing the wastewater treatment sector, food and biotechnology industries, aromatic compound recovery, petrochemical industry, separation of hydrocarbons and removal of toxic organics from industrial effluents.

The membrane forms an integral part of the process. Characteristics of the membrane have a huge influence on the overall performance of the process. Some of the characteristics required for an efficient membrane in the pervaporation process to meet the industry’s demand are it should permit a high flux, offer high selectivity, possess good mechanical, chemical and thermal stabilities as well as be economical. There exist mainly three types of membranes, i.e., inorganic, organic and mixed-matrix membranes (MMMs) that capture the plethora of membrane materials offering often most, though not all, of the required features discussed above. It is however imperative to mention that a particular membrane may not fall entirely in a category, as each of these materials have undergone immense modifications, employing favorable features to be able to propose a membrane best suited for pervaporation. Inorganic and the organic variants have been extensively studied, however, an accurate transport model describing the exact transport mechanism still lacks empirical exactitude.

Organic membranes, constituted predominantly of extensive cross-linked polymer chains, were the first to be investigated. Although organic membranes have an advantage of their easy manufacturing ability, however, they have several disadvantages such as susceptibility to swelling in organic mixtures, dense polymer network, less resilience to temperature, high energy input and a low flux thereby limiting their practical usage. These membranes require rigorous additives to ensure their stability against a wide spectrum of feed concentrations. The additives and stabilizers further complicate the fabrication procedure and increments their production costs for large scale usage.

On the other hand, the inorganic membranes can resist harsh chemical environments thereby extending their scope of operation to wider pH range of organic mixtures, exhibit high thermal stability ensuring high temperature operations, are more resilient towards mechanical failure thus withstanding high transmembrane pressures and are not susceptible to chemicals or swelling in aqueous organic feed mixtures. Alumina, titania, zirconia and silica membranes and their corresponding variations as well as their hybrids constitute this class of membranes. Silica membranes have been found to be better suited for molecular sieving operations due to their successful manipulation to ultra- and microporous amorphous thin layers.

Silica membranes, since their inception, have proven to be apt for pervaporation separation or dehydration as well as gaseous mixture separation. This is largely due to their associated abundance of raw material and easy disposability. In contrast to the various competing alternatives such as mixed matrix membranes, which constitute a relatively new field of research and organic membranes, which suffer from various disadvantages thereby reaching saturation in terms of scientific investigation; silica membranes are posed to serve viable and practical applications for large scale industrial exploitation and this is mainly due to the well-established manufacturing techniques, easy characterization and a deeper understanding of the underlying submicroscopic transport phenomenon. However, it is common knowledge that silica membranes are plagued by many disadvantages, mainly due the inherent nature of the material functional properties, an inadequacy in the complete understanding of the governing mechanism as well as compromised structural integrity towards dilute aqueous organic mixtures.

As the fields of membrane synthesis and pervaporation based separations of solutions have both matured to a large extent, there have been many works that have provided comprehensive insights. The work of Boffa et al. [4] has highlighted both pure silica as well as its modifications pertaining to both the inorganic and organic variety. The work brings into focus the plethoric variations of membranes, which can result from selection and manipulation of materials, however, the practical applications are remiss. The ability of fine tuning the morphology and other physical properties of the sol–gel synthesized silica membranes have been covered in detail by Nagasawa et al. [5]. The versatility of the silica membranes and their suitability for affecting pervaporation-based separations pave a way for future advancements towards obtaining higher fluxes as well as selectivity.

In this review, a comprehensive scientific literature survey on silica membranes, in particular towards their applications related to pervaporation separation of aqueous organic mixtures, solvent recovery and other related avenues are presented. A critical analysis will be done to gauge the viability, evaluate the plethoric variations of silica membranes based on their performance and the current scientific knowledge surrounding the pore localized enigmatic transport mechanism. A comprehensive overview of the structural and material properties, manufacturing techniques as well as the favorable features and limitation in the contest of pervaporation separation and solvent recovery will be presented. Silica membranes have a great potential to successfully boom in the industry and the common consumer market for their wide array of applications stemming from the abovementioned main objectives.

## 2. Manufacturing Techniques

### 2.1. Traditional Methods

There exist many methods for manufacturing silica ceramic membranes, particularly suited to the purposes of pervaporation. One way is via chemical leaching [6]. A highly dispersed, homogeneous matrix of two different phases is prepared. This is then acted upon by a suitable acid, leaching away the dispensable phase and leaving a well interconnected porous structure. This is commonly done for glass, where one phase is sodium borate and the other is the silica rich phase. The structure is strengthened by subsequent heat treatment. In other cases, upon leaching, the glass was subjected to TiCl_4_. TiO_2_ was obtained on submergence in water and then upon light sintering treatment yielded pore volume lesser than 35% with typical pore diameters of 3 nm. Uniformity on pore size distributions was ensured on treatment with plasma containing fluorine after leaching, yielding pore sizes of 100 nm. Sintering is another simplified method where colloid sized particles of alumina, silica, titania and/or zirconia are well mixed in each other. The mixture is then compressed to achieve coagulation followed by calcination and heat treatment to preserve and improve the structure’s mechanical stability [6].

### 2.2. Solution-Gelation Technique

Perhaps, the most suitable and widely performed method of manufacturing pervaporation-friendly silica membranes is via the solution–gelation technique, or otherwise commonly abbreviated as the sol–gel technique. The advent of this technique was brought on through early investigations by Ebelman et al. [7] and Graham et al. [8] on silica sols. The synthesis of ceramic oxides of aluminum, silicon, titanium and zirconium, unobtainable by using the old ceramic powder methods by Roy et al. [9] sparked renewed interest in this method of fabrication. The associated low-processing temperatures, higher purity and enhanced homogeneity have favored the sol–gel technique over other methods. The fine characteristic and structural properties such as size and morphology of the powdered silica spheres can be manipulated by varying the concentration of water and ammonia, the nature of the alkyl member of the silicon alkoxide, the various homologous members of the alcohol family as well as the reactant temperature. There are mainly three routes in the sol–gel process, (1) gelation of a solution of colloidal powders, (2) hydrolysis and polycondensation of alkoxide or nitrate precursors with subsequent hypercritical drying and (3) hydrolysis and polycondensation of alkoxide precursors with subsequent aging and drying at ambient conditions.

Solid particles can be categorized as colloids when their diameters fall in the range of 1–100 nm [10]. The finely divided colloidal particles when dispersed in a continuous liquid phase result in sols. Gels on the other hand are an interconnected rigid network resulting in submicron pores and micrometer long polymeric chains. However, this definition encompasses a plethora of materials, which can be systematically organized in four main categories: (1) well-ordered lamellar structures; (2) covalent polymeric networks, completely disordered; (3) polymer networks formed through physical aggregation, predominantly disordered and (4) particular disordered structures [11].

The main steps that make up the sol–gel process is described in brief to present an overview of the process. The process initiates with mixing of colloidal particles in the aqueous phase, at a pH range inhibiting precipitation for route 1, whereas, routes 2 and 3 entail the hydrolysis of silicon alkoxide comprised of either methyl, ethyl or the propyl group as the alkyl member. This is then followed by a condensation reaction resulting in Si-O-Si bridging bonds between the silica tetrahedral. More interconnectivity is followed by the polycondensation reaction resulting in a SiO_2_ network. The other products of the reaction, which are water and alcohol reside in the pores of the so formed intermittent network. These reactions generate numerous colloidal particles, which result in a low-viscosity liquid amenable to casting using a mold to prevent gel adhesion. After some time, there is a marked increase in the viscosity due to the linking of the colloidal particles. A three-dimensional network, in the shape of the mold, results. Syneresis or aging refers to the immergence of the so formed structure in a liquid. Polycondensation continues to occur as well as reprecipitation. An increase in the thickness of the bridges adds mechanical strength accompanied by a decrease in porosity. To get rid of the liquid phases, drying is ensued. This step needs to be carefully controlled as capillary stress can cause cracks in the gel leading to catastrophic failure. Route 1 exploits the liquid surface energy decreasing tendency of surfactants. Route 2 targets the elimination of small pores via hypercritical evaporation. Tuning the rates of hydrolysis and condensation for yielding monodispersity in pore size comprises route 3. Removal of surface silanol groups is vital for ensuring chemical stability of the structure. The last step involves heating to promote densification, wherein this causes partial elimination of the pores [12].

The silica sols prepared in this manner can then be deposited on substrates to result in thin membranes. This can be achieved by employing widely known techniques such as dip-coating, spin-coating and slip-casting [13]. Silica gels with a structure reminiscent of polymeric gels result, if the hydrolysis rate exceeds the condensation rate, typical of acid catalysis. Denser colloidal silica gels are obtained for basic catalysis, which causes an opposite order of magnitude in the rates. Thus, by a meticulous control over the mechanism and kinetics of the chemical reactions in the sol–gel process, accompanied by well arbitrated use of precursors, surfactants, acid or base catalysis, aging, washing and drying parameters, silica membranes of the desired functional properties can be obtained [14]. It is a well-known fact that sol–gel derived microporous silica thin film membranes demonstrate excellent suitability for gas-based differentiation. However, its inherent unsatisfactory hydrothermal stability and susceptibility to densification are a major challenge. Organically modified silicates present a viable option devoid of these unfavorable features and possess low surface diffusion coefficient and profound crack resistance. The extension of silica membranes particularly to pervaporation separation or dehydration applications was made possible practically by the work of researchers at Energy Research Center of the Netherlands (ECN). The membranes successfully performed dehydration of butanol-water mixture at 95 °C consistently for a period exceeding 18 months [15].

### 2.3. Chemical Vapor Deposition

Another method to obtain defect-free, homogeneously dispersed amorphous silica membranes for the purpose of pervaporation is chemical vapor deposition. There are some variations of this method and each of them offers certain advantages unattainable by other alternatives. It can be classified based on the way precursors are supplied resulting in co-current and counter-diffusion chemical vapor deposition. The basic process involves the thermal degradation of a precursor and its subsequent reaction requiring an oxidant in the gaseous form. The gas needs to be in the vicinity or in direct contact with the substrate, which has been chosen for the said deposition. Tetraethyl orthosilicate or tetramethyl orthosilicate are transported via carriers such as nitrogen or argon. Dioxygen, ozone or nitrous oxide are often chosen as the oxidants. In co-current chemical vapor deposition, one side of the substrate is exposed to the precursors and the opposite side is maintained at the vacuum. The variation in the reactant flux can have a direct effect on the pore size, which in turn determines the permeability as well as selectivity, as accounted for by gas permeance data. In counter-diffusion chemical vapor deposition, the reactants are introduced on both the faces of the membrane, this results in the deposition of silica in the pore walls inside the membrane rather than being present only in the neighborhood of the pore entrance. The technique utilizing atomic layer deposition overcomes the dependence on high temperature synthesis conditions, which result in high costs. Both variations come under the thermal chemical vapor deposition route. Plasma enhanced chemical vapor deposition is another diverse route employing even lower temperatures, no liquid wastes and a higher deposition rate. The last feature makes it suitable for industrial applications. However, the sol–gel technique has proven to be versatile, economic for batch scale processes and can be used to synthesize silica membranes, which lack the inherent hydrophilic nature thereby overcoming this long-standing limitation [13].

Hence, the sol–gel technique is a perfect fit for fabricating silica membranes targeting solvent recovery. The membranes synthesized using this method offer reduced energy consumption in comparison to distillation, reduced operating costs that balance the high installation costs and expeditious performance with characteristic efficiencies for various organic–water mixtures.

## 3. Structure

**Figure 1:**
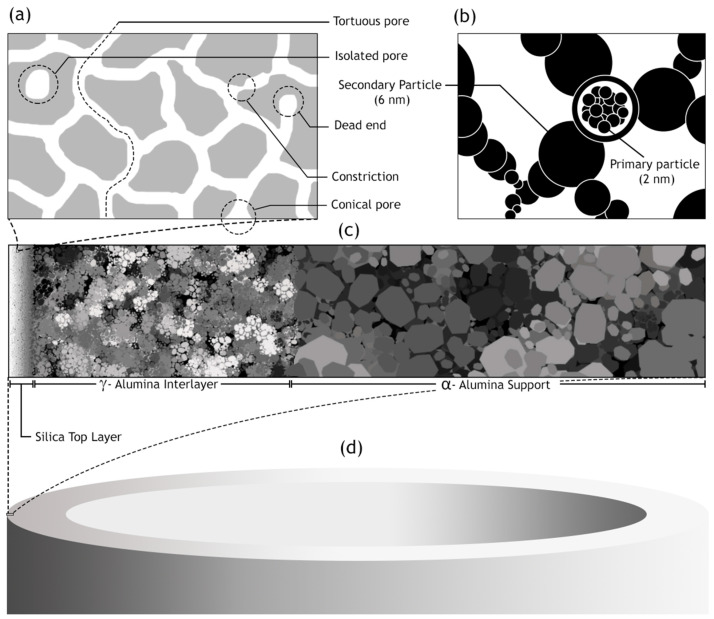
(**a**) Inset of the silica top layer depicting the various pore topologies and the associated nomenclature. (**b**) The agglomerated primary particles resulting in secondary particles, which are the building blocks of the porous network. (**c**) Inset of silica top layer supported by the γ-alumina interlayer and the α-alumina bas layer representing a typical tubular pervaporation module. (**d**) Illustration of a typical tubular pervaporation module.

It is essential to study the structure of the silica membranes obtained using the versatile and extensively investigated sol–gel method of preparation. There is a strong dependence of the pervaporation performance on the structure. The structure of the membrane in-turn is dependent on a complex matrix of chemical and thermal parameters that have a strong impact on the resulting membrane structure. The sol–gel method involves mixing of silicon alkoxide, in water resulting in hydrolysis and condensation yielding Si(OH)_4_ tetrahedra. As a result of the condensation reaction, gradual phase change begins to occur at the molecular level. There is a subsequent change in the viscosity of the mixture, characterized by casting and gelation processes. The nature of the molecular network begins to take form at the gelation phase. However, the gelation stage is an outcome of the hydrolysis and condensation reactions. The main factors are temperature, concentration of the accompanying acid or base and the alkyl member of the silicon alkoxide, which essentially form the kinetics of the hydrolysis and condensation reactions. Establishment of methods to control the reactions and understanding of the underlying mechanism aids in the manipulation of the process to achieve ultra-structure processing. The controllability of these factors is complicated with the simultaneous occurrence of both the reactions. Major steps comprising of the sol–gel technique and their role in determining the structure of the resulting membrane have been described (Figure 1). This is followed by a brief discussion of structure related terminologies used in describing the membranes.

### 3.1. Mixing

**Figure 2:**
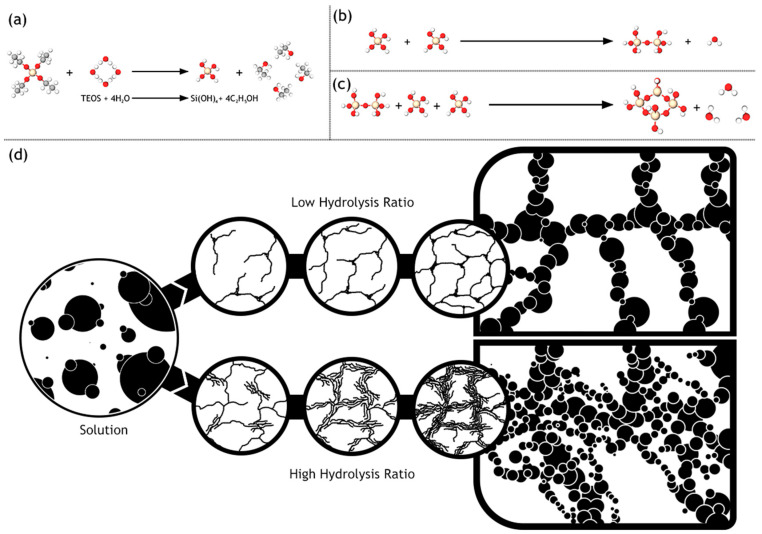
(**a**) Ball and stick model representation of the mixing reaction giving rise to the Si(OH)_4_ tetrahedra. (**b**,**c**) Ball and stick model representation of the polycondensation reactions resulting in cyclic species. (**d**) The role of the hydrolysis ratio in determining the structural characteristics of the resulting porous membrane upon the commencement of gelation.

The hydrolysis of tetraethyl orthosilicate, which yields Si(OH)_4_ tetrahedra, was found to have a linear dependence on the concentration of H^+^/H_3_O^+^ species in acidic medium or OH^−^ species in basic medium based on a study by Aelion et al. [16]. The type of solvent also impacts the rate of hydrolysis with acetonitrile having the highest rate constant and formamide having the lowest, according to the nuclear magnetic resonance studies conducted by Artaki et al. [17]. The molecular weight and length of the alkoxy member of the silicon alkoxide also influences the hydrolysis rate constant, with the heavier and longer ones possessing lower magnitudes (Figure 2). The strongest factor influencing the rate constant is the concentration of the acid or base used as a catalyst. Condensation reactions on the other hand may yield a monomer, dimer, cyclic or straight chain trimer and other higher species. This indicates that there is a simultaneous reverse reaction occurring, evidenced by the presence of monomers as products of depolymerization. There is marked decrease in the reverse reaction in acidic alcoholic solutions, hypothesized, according to Iler et al. [18] to be a result of the inability of siloxane bonds to undergo hydrolysis. As hydrolysis and condensation reaction occur simultaneously in the chemical mixture as summarized by the study of Yoldas et al. [19] it is imperative that the presence of certain species can affect both reactions. H_3_O^+^ in one instance has a constructive influence on the hydrolysis rate constant but OH^−^ has the similar impact on condensation reactions. Extensive studies to identify the intermediates existing prior to gelation were done by Brinker et al. [20]. It was found that an acidic environment favored the production of linear structure due to steric crowding. Basic conditions gave rise to a wider distribution of molecular weights, typical of extensively branched species indicating higher level of cross linking. Subsequent investigations by Orcel et al. [21] conclude that the magnitudes of hydrolysis and condensation rate constants leads to very different scenarios. Linear polymers result as an outcome of expeditious hydrolysis but sluggish condensation, whereas the opposite magnitudes of the rate constants favor the production of heavier and branched polymers. This can be better illustrated with the role of hydrolysis ratio. It is defined as the molar ratio of water to the alkoxide precursor and has an impact on the gelation time.

### 3.2. Gelation

**Figure 3:**
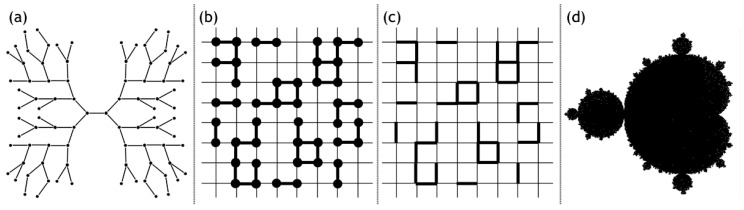
(**a**) Cayley tree or Bethe lattice illustrating the classical or the mean-field theory of gelation. (**b**) Illustration of the simple site percolation model. (**c**) Illustration of the bond percolation model. (**d**) The fractal model as proposed by Mandelbrot.

The transition of a sol, consisting of discrete and minute polymeric phases, to a gel, comprising of interconnected macroparticles is known as the gelation point (Figure 3). The gelation point is highly indiscernible experimentally despite its simple qualitative description. However, as proposed by Sacks and Sheu, [22] a reliable measure is the loss tangent, which is the ratio of storage modulus arising from the elastic component to the loss modulus arising from the viscous component. A marked drop in this parameter with the passage of time gives an estimation of the time taken to reach the gelation point, otherwise known as the gelation time. Gelation time depends on the container size, pH of the solution, size of the alkoxy group, water concentration and temperature.

Various theories exist to describe the phenomenon of gelation. The classical or mean-field theory proposed by Flory et al. [11] establishes a certain assumption to capture the process of gelation in an empirical framework. It is assumed that for a z-functional monomer, the reactivity of the bonds is unaffected even after reaction at a particular site, which is however, not true. It is also assumed that the polymer growth is achieved in congruence to the Cayley tree or Bethe lattice model. This omits the occurrence of closed paths thereby making loops and rings non-existent in the gel matrix. Based on these assumptions the classical theory is able to explain the non-monotonic trend of molecular weight, increasing up to the gel point and then subsequently decreasing. The second assumption however, leads to glaring inadequacies in the proposed theoretical model as it is found that the mass of the polymer is directly proportional to the fourth power of the radius, which implies that density increases linearly with the radius. These dependencies are physically redundant and hence this description was abandoned in favor of the percolation theory.

Percolation theory allows for the formation of closed loops, constituted of various numbers of monomer members. It can be explained by a pictorial representation of a square lattice, where each intersection can be filled randomly. Two adjacent filled intersections give rise to a bond. However, the filling of the lattice by monomers, i.e., bond percolation as opposed to site percolation, is more appropriate. Computer simulations for other more complex lattice frameworks can be carried out to obtain a percolation threshold or size distribution. While percolation model seems to undo the gross deficiency of the classical theory, both are equilibrium models and are inadequate as gelation is a dynamic phenomenon [23].

The fractal model presented comprehensively in the essay by Mandelbrot [24] explains structures encountered in nature, which seem to lack an exact geometrical description. The growth and establishment of a stable gel structure is highly statistical in nature pursuing highly complex patterns. To comprehend this process, it becomes essential to identify a feature that is invariant vis-à-vis the scale of the observation chosen. Mass fractals should be such that they cause a decrease in the density with respect to an increase in the size. Objects explained using the fractal description possess different fractal dimensions as a basis of their characterization. Linear structure has a fractal dimension between 1 and 2, whereas rough structures vary between 2 and 3 with uniform non-fractal objects having their fractal dimension equal to 3.

Small-angle X-ray scattering is a method of characterization that is empirically connected to the radius of gyration of the fractal particle with the scattering intensity. There exists a primary particle (1–2 nm in diameter) constituted of rings or chains of 3 or 4 silica tetrahedral [21]. The gel structure can be envisioned as a three-dimensional network of secondary particles (6 nm in diameter), which in turn are composed of at most 13 primary particles. The Derjaguin–Landau–Verwey–Overbeek theory explains the aggregation of these particles. It postulates that rates of aggregation falls exponentially with increasing size. Higher rate of aggregation is expected for a smaller particle coalescing with a larger one. These primary particles can agglomerate at random sites akin to percolation cluster or via random walk obtaining a seed cluster reminiscent of diffusion-limited aggregation. Secondary particles arise by either of these two mechanisms, which in turn form the gel structure. Application of the Keefer fractal model constrains the fractal dimension from 1.6 to 2.4. Pore radius decreases directly as the fractal dimension increases.

It is conclusive based on investigations carried out using small-angle X-ray scattering, Raman spectroscopy and the Mo dissolution technique that the primary particles composed of closed structures of silica tetrahedral are the building blocks of the membrane. West et al. [25] employed the intermediate neglect of the differential overlap molecular orbital model to calculate the energies of two configurations of silica tetrahedral. It was found that chain structures are more stable than rings for fewer members of the silica tetrahedral. It is hypothesized that 1–2 nm sized primary particles give rise to 4–6 nm sized secondary particles. These secondary particles then undergo further modifications subject to post gelation processes such as drying. According to the quantum mechanical calculations by Yoldas et al. [26] although chains are more likely to form at the initial stages of hydrolysis and condensation reactions, rings consisting of four silica tetrahedra are more likely to form. This is in direct congruence with the presence of 1–2 nm diameter particles.

### 3.3. Aging

The structure of the silica gel continues to change even after gelation. The period after gelation is known as aging and can be categorized into four distinct processes. These may occur at different instants or simultaneously. These are polycondensation, syneresis, coarsening and phase transformation. Polycondensation reactions continue to occur due to the presence of a large amount of silanol groups. In addition to these, there are many silica species that are only bonded to two other silicon atoms and with the passage of time, three and four bonded species increase. The expulsion of silanol groups leads to the formation of newer bonds increasing the cross-linking. Temperature can increase the kinetics of this process. Syneresis refers to the removal of the liquid phase from the pores of the gel and the resulting shrinkage of the gel [26]. There is also a drive to reduce the solid–liquid interfacial area leading to the reduction in size [20]. Vyotsii et al. [27] found that the rate of syneresis falls with respect to time. Ponomareva et al. [28] show that higher temperatures can lead to the gel losing flexibility faster and this prevents the shrinkage to a large extent. Coarsening is said to occur when dissolution and reprecipitation processes occur simultaneously causing a reduction in the surface area. This process is irreversible and is also known as Ostwald ripening. The preferential solubility of convex surfaces in contrast to concave surfaces leads to the growth of neck diameter and a reduction in particle size. This leads to an enhancement in structural strength but a reduction in pore size. Based on the extensive studies conducted by Iler et al. [29], further changes can be affected in the gel structure by a control of parameters such as temperature, pH and the time of exposure to achieve greater strength without altering the pore structure or increase the pore size and decrease the surface area also known as coarsening. Sheinfain et al. [30] found that as the aging process is prolonged, the pore diameter increases and there is less shrinkage. However, when the gel is subjected to further aging; the pore size increases at the cost of a decrease in the surface area. The most common route for gel formation is by the use ofacid-catalyzed silicate precursors. Such kinds of gels are very sensitive to the pH at which they were synthesized and the pH of the water with which they finally washed before drying. Aging also encompasses the time taken to remove the pore liquor by washing. It was also found out that low pH levels increase the pore volume without causing any change in the surface area but exposure to higher pH levels can increase the pore radius but decrease the surface area [31]. Inorganic gels when subjected to a load undergo instantaneous elastic strain and continuous viscous deformation reminiscent of viscoelastic fluids. Prolonged aging implies more degree of condensation reactions leading higher cross-linking and hence, lesser chances of cracking [32].

### 3.4. Drying

Drying of the gels is an integral part of gel formation and can possibly lead to a mechanical failure of the gel if not carried out carefully in a controlled manner. As a result of this it has been studied in a detail by Sherwood et al. [33,34,35], Keey et al. [36], Mujumdar et al. [37], Moore et al. [38] and Scherer et al. [39]. Drying is widely acknowledged to consist of three different stages. Stage 1 can be quantitatively characterized with the evaporation rate per unit area being independent with respect to time and hence known as the “constant rate period”. Consequently, the decrease in the gel volume equals the volume of liquid lost via evaporation. Capillary forces act on the gel structure to cause shrinkage and this stage is said to terminate when there is no further shrinkage. This is succeeded by a stage 2 wherein due to the shrinkage in stage 1, the gel’s strength has increased due to higher packing density. This makes it unsusceptible to any further shrinkage. A critical point ensues wherein the contact angle becomes zero leading to a maximum in the capillary pressure. This causes the liquid to vacate the pores, moving to surfaces that are capable of evaporation. The capillary pressure gradient is the driving force for this fluid transport [37]. Quantitatively, the rate of evaporation decreases. This stage is also the most crack prone period. Diwedi et al. [40] found that for membranes having thicknesses under 40 µm are resistant to cracking. This is because Brinker et al. [41] found that the resultant tensile stress arising from a spatial difference between the shrinkage rate is directly proportional to the thickness of the membrane. Stage 3 occurs when the pores have emptied to the limit where liquid film on the pore walls begins to disappear. Evaporation now occurs within the pore and liquid diffuse to the outer surface in the vapor phase. Diwedi et al. [40] found that 87% of the liquid present in the pore’s escapes followed by 10% in stage 2 and 3% in stage 3.At this stage, the outermost surface has a high presence of silanol groups, which impart a hydrophilic character to the gels. Chemical stabilization ensures the reduction of these silanol groups to reach a critical level to prevent rehydroxylation when in operation. Thermal stabilization leads to decreasing the surface area of the gel to diminish or remove the temperature sensitivity of the structure.

The process of synthesizing the silica membrane encounters these facets invariably. A brief overview of these processes was undertaken as the comprehensive understanding of the silica membrane structure is not possible without it. These processes have a profound impact on sculpting the properties of the membrane, which consequently determines the pervaporation performance.

## 4. Mechanism

A thorough understanding of the mechanism by which permeation across the membrane is achieved, must be undertaken. This not only helps in designing optimized membranes for a separation process for a particular organic component, but also paves the way for overcoming the limitations of a particular design. Since the premise of the paper revolves around only pervaporation-based applications, mechanism governing gas separations will not be discussed. It has been found that the governing model for mass transport is a function of the pore size. It becomes important to categorize pores systematically based on their dimensions. Pores of having diameters larger than 50 nm are known as macropores. Pores possessing diameters in the range of 2–50 nm are known as mesopores. Pores having dimensions lesser than 2 nm are broadly known as micropores. Micropores can be further classified as supermicropores having dimensions greater than 0.7 nm and ultra-micropores with dimensions lesser than 0.7 nm. These size-based categories have been proposed based on the International Union of Pure and Applied Chemistry (IUPAC)nomenclature [13]. Inorganic membranes, even though they have had investigations into their underlying mechanism preceded by their polymeric counterparts, are not subject to ambiguity due to the susceptibility of swelling in the latter [42]. As the pore sizes reduce, it is assumed that phase change ensues wherein the components permeate in the gaseous phase.

There have been many attempts to devise a theoretical model to explain the mechanism of mass transport through the membrane. However, as explained by Bettens et al. [43], the nature of the membrane whether polymeric or inorganic needs to be considered due to the vastly different interactions arising from their material properties. The role of other layers, such as the support, and their ensuing contributions to the transport resistance need to be quantified. As mass transport is involved, concentration polarization in the phase remote from the membrane also requires to be accounted for. The operating parameters are not constant, especially during the initial stages of pervaporation and hence the steady state does not exist, models capable of analyzing these states are preferred. Industrial effluent streams are seldom composed of binary components, and even binary mixtures show interactions between the two phases, affecting the flux and hence these also need to be identified and accommodated. The interval of the concentration chosen for modeling is also important as the relation between various parameters change with a change in feed compositions. A brief discussion on the main theoretical models, proposed by various studies, needs to be undertaken. In the case of interest, i.e., ceramic membranes, adsorption–diffusion theory is studied in detail, which is considered to be composed of three successive steps: (1) sorption of the species onto the membrane surface from the feed, (2) diffusion of the species across the membrane and finally (3) desorption from the membrane into the permeate.

The first step involves the convection assisted mode of transport, wherein selectivity arises from the difference in their speed. The permeating species are further differentiated at the boundary layer, where diffusion assisted mode of transport becomes the dominant mechanism. The diffusion of the faster species is affected by hydrodynamic conditions as well as the geometrical profile of the membrane. Here the influence of Reynolds number becomes important, as highlighted by the studies of Psaume et al. [44] and Cote et al. [45]. An increase in the Reynolds number led to an increase in the total mass transfer coefficient. Numerous studies point out that the presence of concentration polarization influences the transfer coefficient and must be taken into account when setting up a pervaporation module.

The subsequent step to follow is the permeation through the membrane. Two types of models are proposed, which can be classified as: (1) models based on Fick’s binary diffusion equation and, (2) models based on Maxwell–Stefan theory. The linear relation between the flux of the diffusing species with average mixture velocity and the corresponding composition gradient is the statement of Fick’s law of binary diffusion. It is further assumed that the permeating species travel as vapors when traversing through the membrane. The driving force is basically thought to arise from the trans-membrane partial pressure difference. The flux is an Arrhenius or exponential function of the temperature. The solubility and Fickian diffusivity are a function of concentration and interactions between the permeating components are the major cause of deviations from the Henry’s law. The governing equation arising out from Fick’s binary diffusion equation is apt at modeling binary mixtures. However, for multiple feed compositions, the above model is not able to accurately predict the fluxes. Even binary mixtures are explained as a set of three components, which are the species themselves and the membrane, in the adsorption–diffusion models based on Maxwell–Stefan theory. The permeation of a species is thought to arise from the supporting driving force and the opposing frictional effects originating from its interactions with the other component and the membrane. Pervaporation experiments with methanol–water and ethanol–water mixtures require the models based on Fick’s diffusion to undergo diffusion to take into account the interactions between the two components, otherwise also known as coupling effects. However, even with these alterations, the drag effect on the alcohol molecules by water cannot be accommodated. The Maxwell–Stefan models have precedence based on providing a better means of quantification of the drag effect.

An appropriate understanding of the other mechanism is also important. For instance, due to the presence of large pores, typically 20 nm or greater, convective flow is said to occur, which is modeled usingtheHagen–Poiseuille or Kozeny–Carman relation. The flow is said to occur in this regime, according to the Hagen–Poiseuille relation, when the product of volumetric permeability and viscosity is a constant, independent of the nature of the solvent or the operating temperature [42]. This indicates towards the likelihood of defects such as cracks and pinholes, which then need to be remedied or the synthesis steps altered to suit the application. As the pore size is decremented, the interactions of molecules with the walls in the form of collisions happen more frequently than between the molecules themselves. This type of flow is said to be characteristic of the Knudsen diffusion. When the pore diameters approach magnitudes closer to the molecular diameter of the permeating species, then size differences become important. This is known as molecular sieving and is said to occur for pore sizes lesser than 1.5 nm. Selectivity happens exclusively because of the size differences.

## 5. Applications

### 5.1. Advent of Sol–Gel Synthesized Silica Membranes

**Figure 4:**
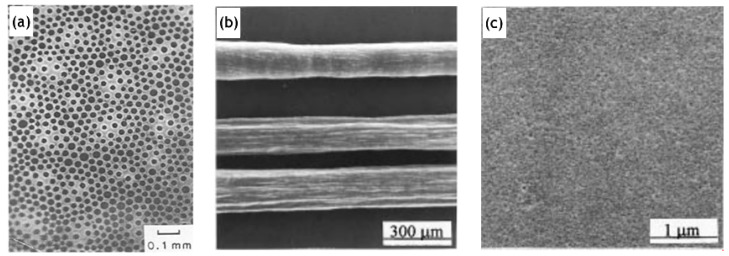
(**a**) Reflected light micrograph of top-side of a 2 month dried sample, adapted from [6], with permission from Copyright (1988), Elsevier Science Publishers. (**b**,**c**) Transmission electron micrographs of ordered mesoporous silica post calcination, adapted from [48] with permission from Copyright (1997), American Chemical Society.

The synthesis of a selective layer for the purpose of pervaporation is itself a complex process. Hence, obtaining a membrane layer devoid of defects, with suitable thickness and porosity was very important. As a result, early studies (Table 1) were quite successful in the synthesis aspect and subsequently laid the foundation for future investigation and modifications. However, the membrane viability requiring verification by undergoing pervaporation could not be covered. In one such study on manufacturing silica membranes by Klein et al. [6], the efficacy and superiority of the sol–gel method was highlighted. The study also emphasizes the high temperature stability and chemical resistance, making them advantageous over the organic membranes. A solution was prepared with tetraethyl orthosilicate (TEOS), water and ethanol. Subsequently 1 M aqueous nitric acid solution was added to initiate hydrolysis. Further treatment with reagents, as well as, ensuing chemical processes; 0.1 mm thick sheets were formed. The amount of water was varied to study the effects on the microstructure. Two moles of water yield the most suitable membrane morphology. The reflected light micrograph of this sample, however, shows connectivity between pinholes on the bottom surface to the large pores present on the top surface. The performance and stability of these membranes were not quantified. This limitation might be largely due to the nascency of the manufacturing process for the fabrication of silica membranes. Pervaporation performance for silica membranes displaying acid resistance was demonstrated by Kitao et al. [46]. The repeated dipping of the porous substrate in silica polymer and calcination resulted in sufficiently small pores (1 µm). The membrane employed a tubular α-alumina substrate as a support. Acetic acid/water pervaporation studies showed favorable fluxes and separation factor in addition to the membrane stability. The membranes synthesized by Kitao et al. corroborate the favorable features of inorganic membranes over their organic counterparts. The study by Brinker et al. [47] demonstrates a major step towards the characterization of membrane parameters. The ultramicroporous membrane synthesized by Brinker et al. has a thickness in the range of 20–120 nm. Alumina supported selective layer facilitates high fluxes. Unprecedented pore sizes, smaller than 10 Å, have the capability of promoting size dependent trans-membrane mass transport at high temperatures resulting in enhanced selectivity (Figure 4). It was also highlighted in this study that particulate sols offer better control to obtain narrow particle size distributions with the porosity being unaffected at 33% for any arrangement, they do suffer many limitations in comparison to the polymeric sols. Particulate sols may crack as the particle size is decreased, tendency for phase transition or grain growth leading to coarse microstructures as well as their intrinsic instability on account of their solubility in liquid phases as the particle size decreases. Polymeric sols do not suffer from these limitations. However, inorganic membranes made using polymeric sols should be thin in order to curb against low volume fraction porosity with a corollary decrease in flux. Three α-alumina layers with anγ-inner layer were used with the pore size ranging from 10 µm to 4.0 nm. It was found that thickness of the selective top layer was dependent on sol aging time, solvent dilution and casting/dipping time.Permeability characterizations using He, N_2_, O_2_ and CO_2_ displayed selectivities in the lower range of Knudsen dominated diffusion regime thereby indicating that the molecular sieving effect was not the major mode of mass transport. This study, however, presented the role of aging and the role of capillary pressure in the drying phase encountered in dip coating. Zeolitic membranes offer profound control over pore sizes ranging between 2 and 10 nm. However, the major hurdle to their adoption as a viable alternative is because of their prevalence existing in a powder-form. Mesoporous silica membranes on the other hand offer controllability over their microstructure. Ryoo et al. [48] successfully fabricated 0.5 mm thick membranes, spanning uptoa few centimeters in size. The membranes were strengthened by repeated adsorption and evacuation of TEOS enhancing strength before calcinations. Cetylpyridinium chloride was chosen as a surfactant for generating mesostructures via S^+^I^−^ type ionic mechanism. Synthesizing ordered mesoporous silica, which was crack resistant during calcination by exposure to tetraethyl orthosilicate vapor at 423 K was a key finding of this study.

As the confidence in the sol–gel technique for synthesis of pervaporation-friendly suitable membranes was established, pervaporation measurements were performed to quantify their performance. Subsequent studies, venturing more into the performance aspects, were also incorporating newer methods of characterizations to ascertain the quality of the membrane. Commercial nanofiltration membranes at that time were polymeric in nature and hence failure at high operating temperatures and instability towards chemicals have limited their usability. Tsuru et al. [56] reported the development of a sol–gel synthesized ceramic membrane made of silica-zirconia composite oxide. The molecular weight cutoff gives an idea of pore size distribution and refers to the ability of a membrane to retain an excess of 90% of a solute in Daltons. The molecular weight cutoff was manipulated by controlling the diameter of the sol used in the final coating stage. As such, this has resulted in four sols with colloidal diameters as 42, 16, 13 and 11 nm. Three membranes were prepared by using the sol with the largest diameters in conjunction with other sols used in the final stage. Characterization using the humid air permeation method yielded pore diameters of 2.9, 1.6 and 1.0 nm in the same order of colloidal diameters of the latter three sols. The addition of zirconia increased the stability of these membranes. The membrane with the smallest pore size showed the permeate volume flux as being dependent on the type of solute, while the others did not show the same dependency. The molecular sieving effect was evidenced based on the filtration performance. Solute-membrane interactions also seem to be in effect due to the difference between the fluxes of sugars, glycols and alcohols. This was later investigated in a study by Tsuru et al. Since the pore sizes were not characteristic of the viscous flow regime, which was demonstrated by the product of permeability and viscosity. Activation energies were found to be a function of the pore size and kinetic diameter of the permeating alcohol. A novel method of preparing mesoporous silica in powder as well as film forms was proposed in the study by Roh et al. [57]. The solvent evaporation method utilizes an acidic environment, constructively influencing the condensation of cationic inorganic species. Powder form was reportedly developed within 10 min, whereas mesoporous films in a matter of seconds. This method avoids extreme synthesis conditions and offers the advantage of recovering the solvent used in the synthesis. However, pore size and stability studies were not characterized for the film-type.

The studies covered in this section by various groups laid the foundation for fabricating silica membranes for the purpose of pervaporation. They also signaled towards the adoption of the solution–gelation route as the most suitable method for manufacturing these membranes. These studies were also monumental as they highlighted the advantages and novel features of the silica-based membranes at a time when the industry and academic research were dominated by the polymeric membranes. The use of silica membranes for pervaporation-based recovery of solvents in contrast to polymeric membranes or distillation was gaining acceptance.

### 5.2. Pervaporation Applications of Commercial Silica Membranes

**Figure 5:**
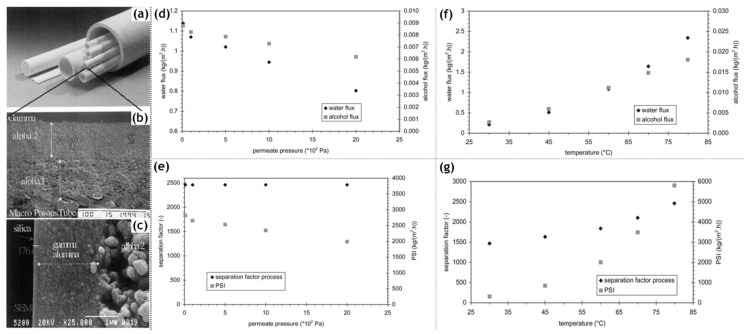
(**a**–**c**) Scanning electron micrographs of the silica membrane developed by the Energy Research Center (ECN), the Netherlands, adapted from [59] with permission from Copyright (2001), Elsevier Science B.V. (**d**) Alcohol and water flux as well as the (**e**) separation factor and pervaporation separation index (PSI), defined as the product of total flux and separation factor for dehydration of isopropanol as a function of permeate pressure (temperature 60 °C, water content 5 wt%). (**f**) Alcohol and water flux as well as the (**g**) separation factor and PSI for dehydration of isopropanol at different temperatures (30–80 °C, 5 wt% water and permeate pressure of 102 Pa). Adapted from [60] with permission from Copyright (2001), Elsevier Science B.V.

Inorganic silica membranes have the immense potential to completely change the industrial processes dependent on distillation. In the late 1990s and the early 2000s, several commercial membranes were being manufactured. These membranes needed to undergo many tests centered on their efficiency, selectivity, flux output and their versatility in dealing with a few possible solvent mixtures. Various alternatives were appearing for silica membrane-based pervaporation [58]. Pervap Silica Membrane System (SMS) was developed by Sulzer Chemtech GmbH (Linden, Germany) in 2000 and is an appropriate example of ceramic membrane suited for industrial pervaporation. Even though, Zeolite A membranes come in the same category, they are highly susceptible to even mild acidic environments and thereby making them unsuitable for effluent streams of esterification reactions as an instance. Other alternatives, offering better mechanical strength as well as stability against extreme pH environments are the various microporous silica membranes fabricated by Netherlands Energy Research Center (ECN) (Petten, The Netherlands). The arrangement of membranes to offer better performances than free standing units is known as a module. The Pervap SMS module has annular passages arranged in series that create turbulence and isothermal operation is ensured. A tubular membrane was developed by ECN, and its pervaporation performance was heavily scrutinized in the study by Veen et al. [59]. The geometry of the membrane was tubular with lengths achievable up to a meter and pores with a 0.4 nm diameter (Figure 5a–c). Extremely high selectivity was ensured as the water flux grew exponentially in contrast to the small organic flux at a temperature ranging from 100–300 °C. Operations at high temperatures require less area, hence balancing the cost of fabrication. The support consists of 2 γ-alumina layers and an α-alumina layer. This was done to ensure a defect-free selective layer of the sol–gel synthesized silica top layer. The feed composition was constituted by 4.5 wt% water, 95.5 wt% isopropyl alcohol and some heavy hydrocarbons. The proposed membranes had better chemical stability than the zeolite A membranes. Pervaporation performances for other aqueous organic mixtures such as acetone, dimethylformamide (DMF), tetrahydrofuran (THF), methylethyl ketone and other common organic compounds were reported (Figure 5d–g). However, the water concentration studied was small enough to avoid stability related complication, due to the inherent hydrophilicity of silica membranes.

Based on the pervaporation of isopropyl alcohol in water using the Netherlands Energy Research Center (ECN) membrane, characterization of the transport mechanism was carried out by Verkerk et al. [60] Maxwell–Stefan modeling considers the interactions between the two components of a binary mixture. These interactions are especially significant for common lower alcohols such as ethanol and isopropyl alcohol (Table 2). Quite simply put, a driving force is envisaged to cause permeation across the microporous ceramic membrane and opposing interactions are thought to arise via friction by the membrane as well as the presence of the other component in the binary system. From the work of Wolf et al. [61], it was found that adsorption does not differentiate between isopropyl alcohol and water. Water flux is caused by its own driving force, whereas the alcohol flux occurs due its driving force as well as the drag force by water. Water flux increased by a large difference, in contrast to the alcohol flux; leading to an increase in the separation factor pervaporation separation index; defined as the product of the total flux and the separation factor. Based on pervaporation experiments, water flux was affected by its own driving force. The presence of isopropanol does seem to cause some disparity between the diffusion coefficient for pure water and water present in a binary system. The flux of alcohol was agreed to the theoretical modeling, however the contribution from the drag effect by water was found to be constant up to 50 wt% water and then increased for dilute solutions.

The study conducted by Constantino et al. [62] demonstrates that the pervaporation process is not limited to only separation based applications. During butyl acetate synthesis, azeotropic mixtures are encountered, which is also a heat sensitive reaction. Both features make it amenable to pervaporation rather than distillation. The commercial tubular silica membrane was utilized based on its effectiveness towards a plethora of organic–water as well as organic–organic systems based on numerous studies. A numerical model was employed using the commercial software gPROMS (general process modeling system). Several binary mixtures comprising of n-butanol and water differing in concentration were studied. Since an increase in temperature increases the driving force for water, the permeate flux experienced an increment. Ternary as well as quaternary mixtures comprising of varying concentrations of n-butanol, acrylic acid, butyl acetate and water were also studied. For multicomponent mixtures, increases in the feed water composition as well as temperature led to an increase in the total permeate flux. It was also observed that the presence of acrylic acid and butyl acetate molecules introduced resistance to the mass transfer process. The applicability of membranes assisted pervaporation towards the esterification reaction was analyzed. A fixed bed reactor was used in congruence with a membrane. This fixed bed membrane reactor resulted in a 66% increase than the reactor for not utilizing membrane assisted pervaporation.

The commercial silica membranes were heavily scrutinized as apparent from their subjection to various aqueous organic mixtures. This was essential in ensuring their suitability for industrial operations. Theses membranes showed promising pervaporation performance as observed by their acceptable selectivity and flux magnitudes. Their inherent structural, thermal and chemical stabilities were displayed in addition to their performance. However, to ascertain their competitiveness from other alternatives such as their polymeric or zeolitic counterparts, comparative studies were required.

### 5.3. Comparative Studies on Commercial Silica Membranes

**Figure 6:**
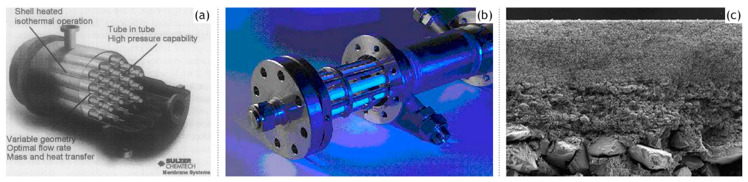
(**a**) Isothermal module concept Pervap^®^ SMS (silica membrane system) from Sulzer Chemtech, adapted from [63], with permission from Copyright (2004), Elsevier Science B.V. (**b**) Bench-scale seven membrane shell-and-tube design module with baffles and perpendicular feed flow, adapted from [64], with permission from Copyright (2005), Elsevier Ltd. (**c**) SEM cross-section of an amorphous silica membrane (scale 1:500), adapted from [65] with permission from Copyright (2005), Elsevier B.V.

Many studies aimed at comparing the various commercial alternatives encompassing silica-based membranes, zeolites as well as polymeric membranes (Figure 6). These membranes were assessed based on their pervaporation performance against a wide matrix of feed compositions differing from each other on the basis of organic components as well as concentrations. These studies aimed at critically assessing the prospect of considering silica membranes as a suitable candidate for affecting membrane based pervaporative solvent recovery and/or dehydration.

The lower energy consumption and almost negligible carbon footprint characteristic of membrane based solvent recovery and dehydration in comparison to conventional distillation are accompanied by the advantage of economic feasibility. Aqueous solutions of 34 different alcohols, glycols, carboxylic acids, esters, ethers, ketones, amines, nitriles and halogenated hydrocarbons were tested on the silica membranes developed by ECN (Petten, The Netherlands) and Sulzer Chemtech GmbH (Linden, Germany) [66]. For the same driving force, fluxes of ketones and ethers were highly satisfactory, whereas glycols ranked the lowest with the rest of the organic families in between (Table 3). The importance of the membrane architecture was realized in this study and forced flows with baffles than an annular duct was found to be a better module design. For the widely applicable case of the isopropyl alcohol and water binary mixture, there was a 40% reduction in cost and 85% energy was saved by switching from distillation to a membrane-based pervaporation unit. Commercially available microporous silica membranes (PERVAP SMS, Sulzer Chemtech GmbH) have been compared with polymeric (PERVAP 2202, PERVAP 2510, Sulzer Chemtech GmbH) and zeolite (NaA type, SMART Cemocal Company Ltd. (Essex, UK)) membranes on the basis of their overall pervaporation performance by Gallego-Lizon et al. [67]. Water and t-BuOH mixtures were chosen with feed concentrations ranging upto 20 wt% water and temperatures in the 60–100 °C range. In the polymeric PVA class of membranes, PERVAP 2510 yielded better flux and displayed greater stability then the other alternatives. Water flux was highly sensitive to temperature variation, especially for dilute mixtures. The separation factor however experienced reduction due to swelling of the membrane. For the silica membrane, the effect of decreasing water concentration on the feed side resulted in enhanced total fluxes and better separation factors. An increase in the temperature increased the flux. Highest fluxes were obtained by the silica membranes followed by the zeolite and then the polymer membranes. The separation factor however was the largest for zeolite, then polymeric and least for the silica membrane. This trade-off needs to be considered for any particular application. Urtiaga et al. [68] sought to compare the pervaporation performances of two commercial silica membranes against the acetone–water mixture (30 wt% water) and other compounds as impurities. Pervap SMS (Sulzer Chemtech, Linden, Germany) and Pervatech BV (Rijssen, The Netherlands) were under investigation. The separation factor for both membranes increased for low water concentrations with an increase in operating temperature, however, SMS membranes were found to be more selective. Templated silica mesoporous membranes offer high porosity, narrow pore size distributions accompanied with low tortuosities [69]. Based on the concentration of the structure directing template as well as the synthetic route, hexagonal (MCM-41), cubic (MCM-48) or lamellar symmetry (MCM-50) pore geometries were reported. Hexagonal MCM-41 type mesoporous silica membranes are not practical as the permeation pathways are oriented perpendicular to the desired direction of mass flow. MCM-48 due to their robustly interconnected porous channels offer minimal tortuosity in every direction, combined with a fabrication strategy to obtain thin membranes, makes them a favorable alternative for pervaporation applications. Commercially available inorganic silica membranes offer more advantages than the polymeric alternatives; however, the correlation of the design parameters with the pervaporation performance parameters is still poorly understood. Further studies are required to completely understand the governing transport phenomenon empirically. The commercial silica membrane known as PVP (Pervatech BV, Rijssen, The Netherlands) was assessed for pervaporation of custom water–isopropanol mixture and a commercial mixture containing the requisite concentrations of the two components [70]. A relation between a flux with a chemical potential gradientis established and verified with experimental results. γ-alumina supported Pervatech PVP and α-alumina supported amorphous silica membrane Pervap SMS were used for the mixtures. It was observed that the water flux increased with increasing temperature and decreasing membrane thickness. The PVP membrane provided a larger water flux than Pervap SMS, however, the mass transfer parameter capturing the influence of adsorption of the permeating species remained constant for both membranes. The importance of process flow adopted in a particular pervaporation process has been highlighted by Schleger et al. [63] Each module is reminiscent of isothermal modules, Pervap silica membranes from Sulzer Chemtech, Linden, Germany, which are constituted of tubes within a tube arrangement with a heat flux entering from the outer surface and pervaporation occurring on the inside. Membranes can undergo series or parallel pathways to suit the needs of the application. The need for finding the best arrangement is governed by the price of the inorganic membranes. An arrangement with less membrane area and higher operating costs is favorable. Table 4 summarizes the performance of Pervap SMS (Sulzer Chemtech, Linden, Germany).

Pervaporative dehydration performance of tubular-type zeolite membranes (Mitsui Engineering and Shipbuilding, Japan) and tubular amorphous silica membranes (ECN, the Netherlands) were compared [64]. These membranes were installed in a bench-scale seven membrane shell and tube design module with baffles perpendicular to the axial stream flow. The feed solution comprised of isopropyl alcohol and water (10 wt%) at 80 °C. The study aimed to examine the impact on pervaporation performance in response to the variation of parameters such as feed composition, operating temperature, driving pressure and state of feed vapor. It was found that with an increase in temperature, both fluxes as well as selectivity were improved. An increase of the water concentration in the feed mixture increased the total flux, and followed the trend opposite to increasing molecular weights for methanol, ethanol and n-butanol in the case of the A-type zeolite membrane. Methanol permeation was found to depend on pore-blocking, which is due to capillary condensation and the adsorption of water molecules on the surface. Amorphous silica membrane yielded a higher water flux for all the three alcohols, but selectivity was slower in comparison to the zeolite membrane. The impact of permeate pressure or the driving force showed different behavior for the two membranes. The water flux was reduced, whereas the selectivity remained the same for the zeolite membrane. Both the water flux and selectivity were reduced with a decrease in the driving force. It is widely acknowledged that these two membranes are better alternatives than polymeric membranes such as PVA/PAN-membranes. Amorphous silica membrane can be described as an excellent flux, medium selectivity membrane, whereas the A-type zeolite membrane can be described as a high flux and high selectivity membrane. The major difference in these characteristics lies in the thickness between the membranes with the forms being 50 times thinner than the latter. The mechanism also differs substantially with silica membrane operating on adsorption and diffusion interactions, whereas molecular sieve effects governing the permeation in the zeolite membrane. Hence, silica membranes are more suitable for separating large amounts of water at the cost of purity. Superheated feed vapor is found to positively influence the performance. Both membranes displayed prolonged stability and ease of manufacturing. Another more extensive comparison of zeolite membranes vis-à-vis silica ceramic membranes was attempted by Sommer et al. [65]. Commercial tubular A-, T- and Y-type zeolites manufactured by Mitsui and microporous silica membranes by ECN and Pervatech were compared, based on their pervaporation performance for more than 30 different mixtures. These encompassed alcohols, glycols, carboxylic acids, esters, ethers, ketones, amines, nitriles and halogenated hydrocarbons as the organic component. Based on these investigations, the selectivity followed the order, zeolite A > zeoliteT > Pervatech silica > ECN silica. The flux, however, follows the order, ECN silica > Pervatech silica > zeolite A > zeolite T. These trends can be explained based on the thicknesses of the two classes of membranes and the ordered structure of the zeolite membranes. All the membranes showed remarkable stability towards aprotic solvents. A-type zeolite is susceptible to acidic compositions, whereas the other zeolite type can tolerate acidity to a certain limit. Silica membranes can remain unaffected to a pH value as low as 3.

An in-depth understanding of the transport mechanism facilitating pervaporation is required to aid in synthesizing membranes properties well suited for the particular application. Various mechanisms through which permeation can proceed have been suggested in the literature; however, the exact mode remains indiscernible. The study by Bettens et al. [71] on commercial tubular microporous silica membrane manufactured by Pervatech (Rijssen, The Netherlands) on pure components such as water, methanol, ethanol, 2-propanol and n-propanol aims to elucidate the transport phenomenon and encounter explicit dependencies of various control and structural parameters on the overall performance. The transport of a pure component has been modeled using the pore flow model as well as the adsorption–diffusion model. The former method assumes convective transport with a phase change from liquid to vapor as the component encounter smaller pores. The latter approach assumes the membrane to be dense so that diffusion and no convection is present. It simplifies the transport to three different regimes such as sorption of the pure component to the membrane surface, followed by diffusion across it and finally desorption on the permeate side to constitute the permeate stream. Pervaporation results indicate significant temperature dependence as evidenced with a water flux being affected the most with temperature variation. This points towards the adsorption–diffusion description of the process. Activation energy of diffusion for water was greater than those of the alcohols. It was found that the strength of adsorption as well as the activation energy increased with the number of carbon atoms for the alcohols. Size parameters such as molecular weight, kinetic diameter and effective diameter had no relation with the flux indicating that sorption was dominant over diffusion and can be the rate determining step for the process. It was found that hydrogen bond interactions affected the sorption majorly and the flux had a strong dependence on polarity. Surface tension and contact angle measurements for alcohols indicate the interplay of poorly understood sublayer interactions. It was concluded that the Fickian analysis for pure component diffusion was apt; however, multicomponent mixtures give rise to many other interactions, which may not fall under its purview. Polymeric membranes possess preferential water selectivity but at the cost of the flux as well as thermal, mechanical and chemical stabilities. On the other hand, inorganic membranes consist of hydrothermally fabricated zeolite membranes and sol–gel synthesized silica membranes. Zeolite membranes are a promising alternative; however, their industrial adaption has been hindered by their high cost of manufacturing. Silica membranes, on the other hand, are a promising alternative as evidenced in their application to water-n-butanol pervaporation as recovery of N-butanol from the extract and raffinite streams in the production of 1,1-dibutoxyethane green fuel. Boutikos et al. [72] investigated the performance of the commercially available silica Pervatech membrane for the above-mentioned application. It was observed that for feed flow rates above 23 L/h, mass transfer resistance due to the presence of the concentration gradient that reduces the flux of the required component was insignificant. The increase in the permeate pressure decreased the flux due to a smaller driving force and marginally decreased the separation factor. An increase in the temperature increased the diffusion coefficient and an increase in the feed water concentration increased the vapor pressure, i.e., the driving force; these work constructively to increase the total permeation flux. The drag effect experienced by the n-butanol molecules as well as their interaction with the water molecules led to an increase in their permeation with increasing water concentration. However, this was accompanied by a decrease in the separation factor. Selectivity did not possess a dependence on temperature. A semiempirical expression was developed for the permeance of water and n-butanol.

Numerous studies on the commercial silica membranes pointed towards them offering acceptable partial component fluxes and selectivities that justified their substitution for the polymeric membranes. Zeolitic membranes cannot be out ruled in comparison to the silica membranes, they do however, face a stiff competition. Commercial silica membranes have displayed impressive chemical resistance against a wide matrix of organic–water mixtures. These membranes are not susceptible to swelling and hence the characterization of the mode of mass transport can be performed without ambiguity. These investigations that sought to compare and quantify the pervaporation performance of silica membranes provided a firm ground for their adoption by industry to achieve solvent recovery (Table 5).

### 5.4. Modified Silica Membranes

Based on the numerous applications presented in the previous sections, the versatility of the silica membranes can be gauged. The inherent material properties provide the synthesized membranes with superior mechanical, thermal and chemical stabilities. The commercial modules have been found to outperform the polymer based membranes. These commercial silica membranes, however, do suffer from several disadvantages stemming out from the same material properties. In addition to this, the commercial silica membranes have fixed membrane structure and hence cause stagnation in the steady growth of silica membrane technology. There exists a large room for improvements to overcome the various limitations of silica membranes. These modifications (Table 6) werecovered in this section as these lay the groundwork for the future scope of this mature and extensively investigated field of research.

It wasobserved that silica membranes didsuffer from their inherent hydrophilic properties. Stability against water is one of the essential features in determining the viability of a particular membrane for its utilization in pervaporation. Silica membranes offer high porosity and flux but however are very susceptible to swelling and degradation on exposure to water whereas crystalline zirconia and titania, although water resistant have poor porosity and size distribution, with pores mainly present on grain boundaries. A hybrid of silica and zirconia or titania can however offer a suitable alternative. The work conducted by Asaeda et al. [2] focuses on analyzing the effect of zirconia concentration and calcination temperature on the stability of the membrane for pervaporation applications. Homogeneity of silica-zirconia composites can be obtained with alkoxide hydrolysis and condensation reactions and subsequent calcination. The sol–gel method entailed using two diverse routes: the conventional partial hydrolysis and condensation reaction or simultaneous hydrolysis and condensation reaction. Hot-coating methods were employed where α-alumina porous cylindrical tubes were used as support, smoothed by α-alumina particles to obtain pore sizes below 1 nm. This was followed by washing in boiling water for guarding against silica rich phases. The SiO_2_-ZrO_2_ (ZrO_2_:50%) membrane was subjected to four consecutive runs against various IPA/water concentrations and it was observed that for each run the water flux drastically decreased monotonically with increasing IPA concentrations. The IPA flux increased and reached a peak at around 20 mol% IPA concentration and then decreased due to a change in the effective pore size brought on the reaction of IPA molecules with hydroxyl groups. It was found that for each subsequent run, preceded by a 12 h submergence in dilute aqueous IPA mixture (4 mol%), the IPA flux increased accompanied with a marginal increase in water flux. This indicated an inherent instability of the membrane hypothesized to be mainly borne out of either intrinsic material properties of the composite membrane or silica rich phases present due to the in-homogeneity resulting from the synthesis route. The former warrants a further investigation into other materials, whereas the latter was taken into account by employing the hot-coating method along with washing in boiling water to remove silica rich phases. Lower heating temperatures yielded high porosity membranes, which has resulted in better performance due to the corollary increase in water flux. Stability against was water improved with an increase in the zirconia content. This delayed the onset of surface hydroxyl groups. Quick drying was favored over slow drying as it ensured well dispersion of the phases. Based on the pervaporation studies conducted on IPA/water and THF/water mixtures, it was concluded that the water flux displayed a dependence on partial water vapor on the feed side rather than vapor pressure difference across the feed and permeate side. A comparative study was conducted by Asaeda et al. [3] between silica, silica-zirconia and silica-titania membranes based on their pervaporative performance against aqueous acetic and propionic acid solutions. The sol–gel synthesis route in conjunction with hot coating methods on a smoothed α-alumina cylindrical tube support were employed for enhanced stability for each of the membrane materials. The sols used were tetra-ethoxysilane, zirconium tetra n-butoxide (ZrTB) and titanium tetra isopropoxide (TiTP). Solubility of silica, zirconia and titania gel powders in deionized water, acetic acid and its aqueous mixture showed marked solubility in silica in water, which decreased significantly with increasing acetic acid concentration. Zirconia solubility increased with increasing acetic acid concentration, whereas titania largely remained insoluble for the entire concentration range. Silica membranes were observed to be quite stable for higher acid concentrations, however, succumbed in the lower concentration range. Silica-zirconia membranes faced degradation, whereas silica-titania membranes maintained structural integrity, but at the cost of low water flux. Silica membranes present a better alternative based on water flux but remained operable for acetic acid concentrations higher than 20 mol%. Simulation results based on the simple pore model were in good agreement with the experimentally obtained results. The inherent instability of silica membranes towards water is a well-known disadvantage. However, this limitation can be overcome by enriching the silica membrane with suitable components or modifications. Brinkman et al. [78] focus on the degradation affected by the hydrolysis of the Si-O-Si siloxane bonds by a substitution with silicon-carbon bonds. This led to a subsequent decrease in the typical pore size due to the smaller bond lengths, thereby making the membrane viable for molecular sieving. This modification also led to a remarkable improvement in its hydrothermal stability. This novel organic–inorganic membrane offers a suitable option for pervaporation applications by constructively combining the positive features of membranes from different classes of materials, which otherwise are not able to fulfill all the desired features on their own. Membrane material selection for a particular application is itself a contentious issue and this is further complicated by a limited understanding of the transport mechanism affecting the required component to diffuse through, ensuring a commercially viable flux level as well as reliable selectivity. In the study by Bettens et al. [74] the simplified Maxwell–Stefan model was vindicated by performing pervaporation of water–alcohol and methanol–alcohol binary mixtures across methylated silica membranes. It was assumed that the diffusion in the bulk of the feed side can be neglected in the cases of turbulence, and only the resistance from the selective layer out of the various supporting interlayers is the rate determining step. Subsequent desorption from the support layer and vapor boundary layer diffusion occur rapidly on the application of low permeate pressure. The generalized Maxwell–Stefan model considers a particular species in a multicomponent system to be acted upon by driving forces and simultaneously encounter friction due to its interaction with other species. Pervaporation of alcohol–water and alcohol–methanol mixtures across an ECN methylated silica membrane at 60 °C and turbulent flow displayed good agreement between the experimentally obtained results and the Maxwell–Stefan single-file diffusion model (Table 7). However, for aqueous mixtures, the dragging of alcohol molecules by the water flux could not be quantified and requires other models such as the extended Langmuir model for complete description.

The phenyl functionalized silica membranes developed by Araki et al. [1] utilizing the sol–gel route of synthesis displayed a prominent hydrophobic nature. The absence of silanol groups compounded the stability of these membranes against dilute aqueous organic feed concentration range. The γ-alumina interlayer in conjunction with α-alumina support provided better control on pore size as well as guarded against defects in the active top-layer. Cetyltrimethyl ammonium bromide (CTAB) was used togenerate pores of desirable size exploiting its molecular template function. The cross-sectional FE-SEM image indicated a 0.1 µm thick silica layer and its remarkable adhesion to the γ-alumina interlayer along with a largely defect-free surface. Intensity of bands in FT-IR spectrum corresponding to the phenyl group was increased with increasing phenyltriethoxysilane (PhTES) concentration. Contact angle measurements corroborated the hypothesis that the hydrophobic character increased with increasing PhTES concentration. This was furthermore accompanied with a decrease in single gas permeances for He, H_2_, CO_2_, N_2_ and CH_4_. Pervaporation studies with ethyl acetate (EA) indicated 0.32 mol/l CTAB concentration as optimum since higher concentrations could not be obtained. EA flux increased with the CTAB concentration, whereas water flux stagnated due to the pore blocking action of EA molecules. EA diffusion was increased with increasing PhTES concentration along with a significant decrease in water flux and 80 mol% PhTES concentration offered the highest separation factor. The separation factor was decreased, whereas the fluxes for both the components increased as a function of EA feed concentration. The temperature of the feed was varied, and it was observed to increase the fluxes of both EA and water, which however led to a decrease in the separation factor due to a larger increment in the latter. The water flux was unaffected by the presence of the functional group and the pore blocking effect was sustained even at elevated temperatures. The partition coefficients for EA, methylethyl ketone (MEK) and isopropyl alcohol (IPA) showed that the flux of the organic component increased in accordance with its hydrophobic character. It was also concluded that a large kinetic diameter required fewer number of molecules to achieve the desired level of pore blocking.

The pervaporation performance is a multifunctionally dependent complex process. As such, there are many parameters to be controlled and multiple trade-offs to balance. The hydrodynamic conditions on the feed side also have a bearing on the permeation transport as evidenced in the study that was conducted by Cuperus et al. [80] on tubular silica membranes. The Reynolds number as a function of the linear flow velocity of the feed and inner diameter of the membrane as the characteristic length was used to find an optimum between the increased water flux and the costs of sustaining high operating feed flow rates. A Reynolds number of 3500 was proposed as a suitable compromise between the two factors. The major limitation to the versatility for handling various mixtures for pervaporation as well as their limited penetration into the industry are because of the instability induced by extreme pH environments. Modifications to the support and selective layer by doping were investigated in the study conducted by Sekulic et al. [81]. It was observed that the addition of alumina negatively impacts the chemical stability, whereas the addition of titania and zirconia in controlled amounts, enhances the range of stable operation. However, high dopant levels come at a cost of poor structural strength due to the degradation of the polymeric matrix.

Pervaporation studies by Chowdhury et al. [69] on these membranes and their comparison with α-alumina supported mesoporous γ-alumina membranes show their versatility as being a possible option for industrial nanofiltration processes. Investigation into permeability was conducted by passing water, ethanol, 1-propanol, 2-butanol, toluene and hexane through both the membranes. In the case of the α-alumina support layer, pressure difference and liquid viscosity are the two main parameters influencing the flux, lying in the domain of viscous flow model, which is typical of macroporous membranes. γ-alumina layers, on the other hand, have the capability of differentiation based on the hydrophobic or hydrophilic nature. Permeation of hydrophilic liquids is favored because of the constructive hydrogen bond interactions. These have a marked impact on the effective pore size available for each of the testing species. The existence of a threshold pressure to affect non-zero flux exclusively for toluene and hexane could not be explained. In contrast to the γ-alumina membrane, Mobil Composition of Matter No. 48(MCM-48) membranes displayed different fluxes for each of the alcohols, with alkoxylation possibly hindering the flow of higher alcohols. MCM-48 membranes were found to be superior primarily because of their lesser thickness and contributions from high porosity and high tortuosity. The effect of mesoporous layer on the overall pervaporation performance and membrane stability was studied by Sekulic et al. [84]. Two membranes differing only in the material constituting the interlayer were examined. In one case was the widely used γ-alumina interlayer, which restricted the operation of the membrane to a pH range of 4–10, whereas in other case was crystalline mesoporous titania. The support was α-alumina with a microporous silica selective top-layer in both the cases. Pervaporation experiments were conducted using 2-butanol, 2-propanol or ethanol and water mixtures. Size based molecular sieving caused a 2-butanol/water mixture to have the lowest flux and highest selectivity with the ethanol/water mixture on the opposite end of the spectrum for both the membranes. Increase in the flux and decrease in the separation factor were demonstrated for dilute aqueous mixtures attributed mainly to the dragging effect by the water molecules. Enhanced temperature operation provided better overall pervaporation performance. There was a marked decrease in the separation factors in the case of mesoporous titania. Single gas permeation results indicated the absence of defects for both the membranes. The main reason for this difference was hypothesized to be due to the trivalent nature of the aluminum cation, introducing acidic sites, making the membrane hydrophilic, whereas the oxidation state of titania being the same as silica does not affect any changes in the otherwise pre-existing membrane. The presence of these hydrophilic sites on and at the vicinity of the pore walls can increase the driving force causing preferential permeation of water.

The substitution of the reactive OH groups on the surface with methyl groups, improve the stability and hence the life-cycle of the silica membranes. This was observed in the study conducted by Campaniello et al. [15]. The stability and long period analysis of membranes still require further studies to be able to assess them for industrial applications. Mesoporous γ-alumina support was employed and a single step as well as two step acid catalyzed hydrolysis methods for sol preparation was performed. The two-step route yielded a narrower particle size distribution in contrast to pure silica and one step route methylated silica membranes. Pure silica membranes showed a marked decrease in the water flux when in operation for few weeks. This affect can be attributed to the pore blocking by the water molecules with more prominent hydrogen bridge or chemisorptions in comparison to physisorption. The methylated silica membranes decrease the water flux due to their hydrophobic nature, however the membranes remain significantly more stable than the pure counterpart. However, even with methylation, operating temperatures above 95 °C has led to degradation of the selective top layer.

**Figure 7:**
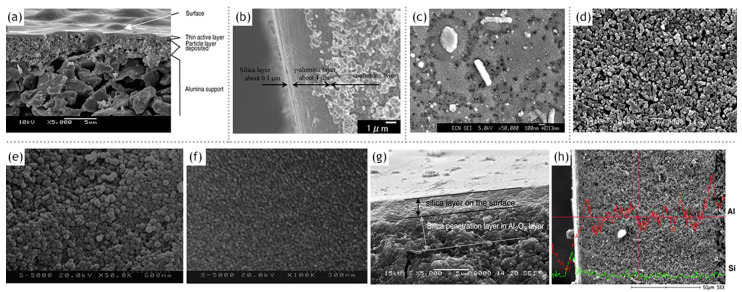
(**a**) Typical SEM micrograph of amembrane cross section, adapted from [2], with permission from Copyright (2002), Elsevier Science B.V. (**b**) FE-SEM image for the cross section of phenyl functionalized silica membrane, adapted from [1], with permission from Copyright (2011), Elsevier Science B.V. (**c**) SEM micrograph of a ‘30% methylated silica’ membrane after testing in pervaporation at 135 °C for 7 days, adapted from [15], with permission from Copyright (2004), The Royal Society of Chemistry. SEM photographs of (**d**) the surface of α-Al_2_O_3_ support; (**e**) the surface of intermediate silica membrane after the first dip-coating and surface modification; (**f**,**g**) the surface and cross-section of the mesoporous silica membrane after the second dip-coating and surface modification and (**h**) cross-section with an energy dispersive X-ray(EDX) line indicating the distribution and strength of aluminum and silicon element. Adapted from [75], with permission from Copyright (2011), Elsevier Science B.V.

The mechanism of mass transport is a function of pore sizes as mentioned in a variety of literature sources; it however is also heavily dependent on the nature of the species being permeated through the membrane. Pervaporation experiments were carried out on water–organic component mixtures by Johan et al. [73]. The organic components used in the study are methanol, *N*,*N*-dimethylformamide and 1,4-dioxane. Each of the components were investigated based on the response of their flux and separation factor to changes in temperature and feed composition. It was observed that because of its large kinetic diameter and small dipole moment, 1,4-dioxane yielded large separation factors. However, the opposite was true, for methanol, which with its quite similar diameter to water and low dipole moment, was able to penetrate the membrane along with water. DMF, on the other hand, has a large diameter and significant dipole moment, which cause it to be adhered to the pore walls resulting in delayed diffusion. As the silica pore size is lesser than the molecular diameter of water, surface diffusion rather than gas diffusion was the governing mechanism explaining water transport. The fluxes for methanol could not be explained by the standard Maxwell–Stefan diffusion theory, as the driving force was found to be constituted of partial pressure gradient as well as the coupling effect of being dragged along with the considerably higher water flux. It was observed that 1,4-dioxane diffusion occurs via the mode of viscous flow through pathways such as mesopores and small defects. DMF is also thought to undergo the same mode of transport although its micropore permeation is also contested. This is because it demonstrated a pore blocking effect, restricted to the microporous domain. The mechanism of pervaporation separation achieved with hydrophobic mesoporous silica membrane has been disseminated from the study conducted by Jin et al. [75]. The silica membrane was prepared by dip-coating on commercial porous alumina tubes. The hydrophobicity was attributed to the membrane by subsequent surface modification using 1,1,1,3,3,3-hexamethyldisilazane (Figure 7). XRD patterns showed that the α-alumina support had been adequately covered by the silica selective layer. Successive dip-coating ensured minimal penetration of the silica sol into the pores of the alumina support. The surface modification resulted in the appearance of trimethylsilyl functional groups, which has rendered both the increased surface hydrophobicity and the surface potential. Pervaporation studies were conducted with binary mixtures of ethanol and acetone with water as well as ternary mixtures comprising of ethanol or acetone with water and acetic acid for mimicking the actual industrial effluent streams. In contrast to zeolites, separation factor was less, however, the corresponding flux was large. The membrane was performed satisfactorily for ternary mixtures as well. The adsorption diffusion model adequately explains the transport model in the study.

**Figure 8:**
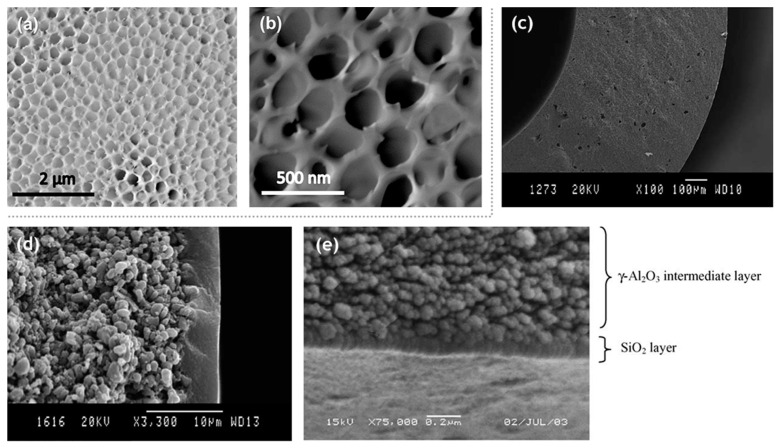
Top-view SEM images of (**a**) anodic aluminum oxide support coated with silica film (70 nm thick) after calcination and (**b**) anodic aluminum oxide support coated with silica film (70 nm thick) after calcination and several minutes of SEM exposure. Adapted from [87], with permission from Copyright (2016), American Chemical Society. SEM micrographs of the various layers constituting the membrane: (**c**) cross-section substrate (magnification 100×), (**d**) cross-section intermediate γ-Al_2_O_3_ layers on top of the substrate (magnification 3300×) and (**e**) cross-section silica membrane on the intermediate γ-Al_2_O_3_ layers (magnification 75000×). Adapted from [82], with permission from Copyright (2004), Elsevier B.V.

Yamashita et al. [85] in their work have tried to overcome the problem of the orientation of microscale channels perpendicular to the direction of intended permeation flow. Porous anodic alumina in conjunction with the silica-surfactant nanochannel assembly membrane was utilized to separate phenol, benzene sulfonate and benzene disulfonate from a water–ethanol solution. The pore sizes in the anodic alumina ranged from 10 to 100 nm, which upon treating with a solution of cetyltrimethylammonium bromide surfactant and tetraethyl orthosilicate formed pores of size 3.4 nm along with the preexisting channels. The removal of surfactant was refrained as this promotes hydrophobic interactions between the channel walls and the organic molecules. Molecular sieving effects can be overlooked due to similar molecular diameters. The three molecules differ in terms of charges, with phenol being neutral, single anionic charge carried by benzene sulfonate and a dianionic charge carried by benzene disulfonate. The flux of the organic molecules followed the trend of decreasing charge due to the anion-exchange adsorption mechanism. A decrease in the ethanol concentration decreased the flux of phenol, whereas it was increased for the other two. This shows the interplay of dipole interactions, solvation and anion-exchange efficiencies and the ability to control the flux by simple variation in ethanol–water composition. The conventional methods of synthesis for silica membranes are mainly sol–gel routes and chemical vapor deposition. Although extensive studies on silica membranes derived from these manufacturing techniques have been conducted, there remain many limitations intrinsic to the nature of each of these processes. A novel method of synthesizing silica microspheres using microfluidic hydrodynamic flow has been proposed by Carroll et al. [86]. This can eliminate the wide pore size distributions and offer opportunities for assembly into two- and three-dimensional arrays with hierarchical porosity. The microspheres were prevented from coalescing by constant stirring. Utilizing this technique, monodispersity of size was ensured. At most 5 µm sized particle can be generated by using channel widths of the order of 10 µm fabricated using soft lithography. Smaller droplets require more meticulous fabrication techniques and/or rigorous flow rates. The orientation and the corresponding tortuosity of the pores are essential in controlling the amount of resistance that a preferred component endures to form the permeate. In this regard, a study by Wooten et al. [87] overcame the parallel orientation of the hexagonal pore structures rendering them highly useful for pervaporation analyses (Figure 8). This required the use of surface modification, surface patterning and control over other parameters. Successful orientation of the hexagonal pores was characterized by transmission electron microscopy of the silicon thin film after the dissolution of the strongly bonded anodic aluminum oxide support, pore visibility in scanning electron microscopy and solvent flux data obtained during pervaporation measurements (Figure 8). The templating was done using sandwiching between neutral copolymer barrier layers or air-curing.

Tsuru et al. [88] aimed to differentiate organic–organic mixtures. The mixtures studied were comprised of hexanol, octanol or decanol in ethanol or hexane, decane or tetradecane in ethanol. Reverse osmosis was employed for their separation from the ethanol medium. A silica-zirconia membrane was employed for this task with pore sizes ranging between 0.8 and 2 nm and a molecular weight cutoff of 200 Da. An increase in pressure has increased the expulsion of all the alcohols and alkanes. Temperature did not affect the alkanes, whereas alcohols showed a decrease with an increasing trend. The application of the bilayer model showed a good agreement between reverse osmosis and diffusion.

Distillation is more suited for scaled up industrial applications but proves to be expensive or impractical in dealing with azeotropes. Membrane technology requires less energy consumption and can differentiate azeotropes, and close boiling point mixtures, but scalability has always been an issue. A combination or a hybrid of these two technologies used in conjunction can offer many viable process flows. These possibilities were explored by Sommer et al. [89]. High temperature operation is always desired and hence membranes can replace distillation columns in spray drying, dimethyl carbonate and methyl-tert-butyl-ether production and many other applications. Four main configurations were proposed, which are as follows: (1) azeotrope splitting by membrane followed by distillation, (2) pervaporation by membrane of top or bottom stream of the distillation, (3) membrane between two distillation columns and (4) membrane combined with column via side stream processing. Configuration 1 was suited for highly volatile acetonitrile and methyl ethyl ketone. Configuration 3 offered a 20% reduction in cost in contrast to the conventional route. Configuration 4 requires a lesser number of trays and reduces the reflux ratio of the accompanying column when the membrane is in proximity to the pinch point. A 40% reduction in the cost of investment and operation as well as 85% savings of energy could be achieved. The incorporation of inorganic membranes rather than polymeric ones offered 11% lower separation costs. A combination of membrane-based vapor permeation and distillation proved to be superior to isothermal and adiabatic permeation based on the overall performance.

γ-alumina interlayers were supported on ceramic hollow fiber membrane supports in the study by Peters et al. [82]. The organic solvents used in pervaporation dehydration were n-butanol and dimethylformamide. Single gas permeation studies showed selectivity towards helium with low permeation for nitrogen, characteristic of microporous silica membranes. The interaction of n-butanol with the surface silanol groups as well as the alumina layers may promote densification leading to a decrease in the performance of the membrane with the passage of time. The selectivity, however, is improved on prolonged exposure due to the pore blocking by alcohol. As dimethylformamide has an inherently large dipole moment, the same kind of results is expected for its prolonged interaction to the silica membrane. The molecular diameter is large and thereby making its permeation highly susceptible to pore sizes. In contrast to the tubular supports, ceramic hollow fibers were found to be economical and possessing large surface area/volume.

Although the hydrothermal stability of silica membranes has posed a marked limitation in their adoption as a versatile tool for industrial applications, the different solutions in mitigating this feature have spurred several membranes. In one such study conducted by ten Hove et al. [90] silica, hybrid silica as well as zirconium doped silica membranes were analyzed on the basis of gar permeance. The latter two have been derived using 1,2-bis(triethoxysilyl)ethane as a precursor. Hydrothermal tests were conducted, which involved placing the membranes just above the meniscus of boiling water while ensuring no direct contact. It was noticed that the membranes showed the same permeance and selectivity as that of the α-alumina layer and thereby indicating the deterioration of the selective top layer. A hydrothermal test performed at 100 °C, resulted in silica membrane losing its selectivity with the other two variants experiencing no change. Subsequently membranes were exposed to conditions akin to the water gas shift reaction. Once again hybrid silica membranes did not display any major changes until a temperature limit of 300 °C. However, exposures to conditions around 400 °C show the indications of degradation of the hybrid and zirconium doped silica membranes. Another peculiar observation was the measurable drop in the carbon dioxide permeance at 200 °C and 300 °C for the zirconium doped variety. This was ascribed to the Bronsted-acidic property of zirconium/silica oxides. The effects of introducing alkali metal cations in the pure silica mordenite framework inverted type zeolite membranes have seldom been studied. The study conducted by Xu et al. [91] aims to investigate the role that this addition may have towards the pervaporation performance of the membrane. Varying amounts of sodium chloride was added and it was observed that this aided in the nucleation process and hence leading to denser and more stable hydrophobic membranes. However, increased amounts of this dopant repelled the agglomeration of nucleation sites thereby leading to a weaker diffused gel structure. The potassium ions displayed a similar trend, whereas cesium ions had a negative effect on the pervaporation performance of the membrane. A 5 wt % ethanol–water mixture was tested at 60 °C. To impart a hydrophobic character to silica membranes, which are inherently hydrophilic by nature, the surface silanol groups need to be removed or shielded to eliminate their interaction with water molecules. Vinyl-functionalized silica membranes have been synthesized and tested for their pervaporation performance in the study conducted by Araki et al. [92]. The membrane architecture comprised of an α-alumina base layer and γ-alumina interlayer and the selective top layer was prepared via the sol–gel route with vinyltrimethoxysilane as the precursor. The vinyl layer degrades at temperatures higher than 453 K. The role of the support layer on the pervaporation performance was also investigated. Two different supports, the first one being the α-alumina tubular support and the other one being alumina hollow fiber. Ethanol-, methyl acetate- and methyl ethyl ketone-water binary systems were subjected to the pervaporation process. The hollow fiber alumina support typically yielded three times the fluxes of the conventional tubular support. The elimination of surface silanol groups to combat the hydrophilic character that leads to structural degradation has been a topic of intense research in the domain of silica based pervaporation membranes. In the study by Kanezashi et al. [93] the silanol groups have been substituted by fluorine. The membrane was synthesized via the sol–gel route, using NH_4_F for introducing the dopant in the silica gel network and bis(triethoxysilyl)methane as the silica precursor. Fluorine manifested itself by forming covalent bonds with carbon and silica atoms. Increased helium and hydrogen permeances indicated a small enlargement of the pore size. The water permeation flux as well as the selectivity remained somewhat unchanged however, the newly synthesized membrane showed a substantial decrease in the hydrophilic character. However, pervaporation for long term operating time intervals need to be performed to ascertain any direct contribution to membrane stability. Another study conducted by Wu et al. [94] presents a solution to a recurring theme in silica based membrane separation. For silica membranes to offer a viable alternative to conventional separation processes in the industry, stability or long-term stable operation is a prerequisite. Pure silica mordenite framework inverted type zeolite or silicalite-1 has uniform pore size distribution and high characteristic selectivity and flux. Immersion in the dopamine solution generated a thin indiscernible poly-dopamine layer. This layer did not impart any measurable thickness as well as any change in the static contact angle. The typical deterioration of pervaporation performance as observed in unmodified silicalite-1 membranes was slowed down for the modified membranes. It was also observed that prolonging the duration of immersion did not yield positive results and hence an optimum time of 24 h was finalized. This study presents a useful modification to silicalite-1 membranes, particularly for ethanol dehydration to produce bioethanol. In the study by Zhang et al. [95] lanthanum and yttrium doped mesoporous silica membranes were assessed for their pervaporation performance in desalination applications. The doping with these rare earth elements can potentially change the hydrothermal stability by establishing strong silicon oxygen and a rare earth element covalent bond. The associated hydrophilicity aided for efficient water transport across the membrane yielding successful desalination at significantly lower operating temperature than the normal boiling point of water. The finer microstructure as well as lower mass transfer resistance also led to the efficacy of the desalination process. The successful up scaling of this method can address the problem of growing shortage of freshwater in the world.

The study by Klinov et al. [96] explores on the validity of the solution-diffusion model and its use for determining the optimum operating conditions as well as the best suited selective layer thickness for pervaporation of ethanol–water and isopropanol–water mixtures. The membrane used pertains to the class of organic–inorganic hybrid silica membranes. The organic component imparting enhanced hydrothermal stability and viscosity to guard against structural failures due to nanocracks. The inorganic component imparts hydrophilicity as well as structural strength. These types of membranes were developed by Pervatech BV, Rijssen, The Netherlands. When the organic component flux was compared for both the binary mixtures, the ethanol system was greater by an order of magnitude from the isopropanol system, the difference being attributed to the kinetic diameter of the molecules as well as the relative volatility of water. The selectivity shows a non-monotonic trend and hence a maximum for a particular water concentration and hence this factor needs to be addressed to ensure smooth operation. The optimum thickness of the selective layer was proposed to be approximately 50 nm. The predictions of the solution-diffusion model are in good agreement with the experimental results.

Modified silica membranes and their proposed applications have now begun to surpass the domain of separation based on membrane assisted pervaporation process. Novel and interesting solutions to a plethora of problems plaguing the conventional technologies are coming up in recent years. Acetone and methyl ethyl ketone are valuable solvents, especially in the pharmaceutical industry. Their successful isolation from binary or multicomponent mixtures provides an invaluable route, bypassing the limitation and high costs associated with distillation and other conventional technologies in use. The study by La Rocca et al. [97] deals with this problem using two variants of zirconium doped hybrid silica membranes. These variants defer in the precursor used, which are bis(triethoxysilyl)methane and bis(triethoxysilyl)ethane. The doping positively affected the selectivity and influenced flux. An increase in the number of undesirable components in multicomponent feed mixtures inadvertently reduced the flux. A drop in the pervaporation performance was observed especially in the cases of toluene and dichloromethane. This was hypothesized to arise out of unaccounted coupling effects. The control of microstructure by the variation in synthesis conditions has a profound impact on the pervaporation performance of a particular membrane. In the study by Lan et al. [98] this aspect of membrane based pervaporation has been explored. However, to establish an unequivocal link between these two parameters, the number of characterizations such as single component gas permeation, water contact angle measurement, scanning electron microscopy, X-ray diffraction as well as energy dispersive X-ray need to be performed as evident in this study. Tetrabutylammonium ion in modest amounts leads to a dense and hydrophobic membrane, which leads to good pervaporation performance and stability. However, higher amounts tended to impede and disrupt the crystallization process, leading to weaker and compromised gel networks. The effect of other tetraalkylammonium ions was also investigated; however, the same constructive effects were not encountered.

Novel methods to establish a circular economy in the ambit of membrane based pervaporation technology are gaining ground in recent years. Due to the abundance and non-toxicity of silica, waste products can act as silica source rather than on the sole reliance on costly precursors. In the study conducted by da Silva et al. [99] rice husk was subjected to acid leaching treatment to isolate silica. The high purity silica was then applied in an organic membrane to improve the pervaporation performance. The importance of pervaporation in industries based on solvents is quite evident. However, the study by Wang et al. [100] brings forth the applications centered on purification and removal of toxic chemicals from the environment. Methyl tert-butyl ether is a toxic compound requiring efficient removal to mitigate the risks posed to the ecosystem as well as counter any exposure to the general populace. Mesoporous silica membranes employing α-alumina support synthesized via the sol–gel route and dip-coating technique was further enhanced for its effectiveness. Silylation of the membranes was done using 1H,1H,2H,2H-perfluorooctyltriethoxysilane, trifluoropropyltriethoxysilane, octyltriethoxysilane and propyltriethoxysilane. There was an increase in the hydrophobicity of the silylated membranes as evidenced by the contact angle measurements. This improved the separation factor for methyl tert-butyl ether/water systems. 1H,1H,2H,2H-perfluorooctyltriethoxysilane silylated membrane yielded highest selectivity, whereas propyltriethoxysilanesilylated membrane yielded the highest flux. The membrane silylated with the former provided a suitable tradeoff between selectivity and flux. Pure silica mordenite framework inverted type zeolite also known as silicalite-1 was synthesized in the study conducted by Ueno et al. [101]. The support used was tubular silica and no other silica source, for instance, synthesis gel, was employed. The source of seeding of the selective layer was the support itself. The membrane displayed a good pervaporation performance for ethanol–water system. A selectivity of 66 and high fluxes were achieved. The selective layer thickness was obtained to be around 7 µm. The negligible use of reagents and the comparatively simplistic fabrication procedure makes it amenable to adoption for industrial applications. In the study by Raza et al. [102] two routes of membrane synthesis and their subsequent impact on the pervaporation performance were investigated. Hybrid membranes were fabricated using bis(triethoxysilyl)ethane with and without hydrochloric acid treatment via the sol–gel route. The subsequent treatment with the acid promoted densification leading to higher separation factors, however, a lesser flux in comparison to the untreated one. However, the drop in the total flux was not significant, when applied to pervaporation of acetic acid-water binary systems. The stability was found to be improved in the long run.

Modified membranes have the potential of overcoming the limitations posed by the α-alumina supported traditional mesoporous silica tubular membrane. Studies aimed at improving the hydrophobic character and the overall stability of the membrane has been the recurring theme in recent times when compared to the long history of this mature field. It is imperative to acknowledge the saturation of information surrounding the commercial membranes. It is imminent that modified silica membranes tailored according to very specific set of conditions offer enormous strides for this ever-growing and dynamic field.

## 6. Influencing Factors on Pervaporation Performances

### 6.1. Effect of Feed Temperature

Early studies were primarily based on optimizing the sol–gel technique for synthesizing pervaporation membranes. Due to this shift in focus, the effect of temperature on the pervaporation performance was not studied as the pervaporation itself, was seldom performed. However, with the establishment of this technique and fueled by the inception of commercially available silica membrane modules, pervaporation performance became an important part of studies to affirm the validity of the synthesized or commercial silica membranes.

**Figure 9:**
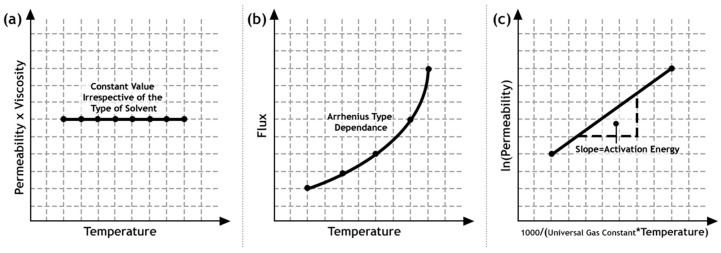
(**a**) The plot between the product of permeability and viscosity against temperature for various solvents depicting the validity of the viscous flow mechanism. (**b**,**c**) The nature of plots obtained when pervaporation is occurring in theregime described by the adsorption–diffusion model.

Extensive studies were done on understanding the effect of temperature over different membranes and the feed mixtures, at the same time by varying the type of organic component as well as the concentrations. It was observed that an increase in the temperature increased the fluxes of both ethanol and water; however, the increase in the latter was far more pronounced. A temperature limit of 240 °C was reported ensuring stable operation. There was also a marked decrease in the required membrane surface area required thereby bringing down material and synthesis costs [59]. An increase in the feed temperature for isopropanol–water mixtures displays a marginal increase in the alcohol flux but a large increase in the water flux. This unequal increase enhanced the selectivity and pervaporation separation index [60]. Both water and t-butanol fluxes increased with an increment in temperature. Selectivity also increased with the temperature increase [67]. Attempts were being made to identify the nature of this dependence and studies carried out are giving only a qualitative description of this relation. Utilizing models based on the solution-diffusion theory have bridged the gap between theoretical and experimental results. It was observed that isopropyl alcohol and water fluxes showed an Arrhenius type exponential temperature functional dependence. This led to exponential trends of fluxes against temperature [66]. Acetone–water mixtures showed the same temperature dependence of fluxes for the permeating species [68]. Water–isopropanol and an effluent stream resulting from synthesis of rubber antioxidants comprising of water and acetone were subject to pervaporative dehydration. The expression for the flux also had an Arrhenius type dependence on temperature [70]. This was evident as the transport mechanism derived from the solution-diffusion model yields an Arrhenius type temperature dependence of the flux of each component with temperature. This exponential growth in the flux with temperature also points towards a decreasing membrane surface area for efficient yields due to high compensatory fluxes. It was also found out that isothermal operating conditions are more suited than adiabatic conditions with respect to flux [63]. The fact that material cost may be balanced was also corroborated another study based on the partial permeate fluxes for water–ethanol and water–isopropanol mixtures. Flux showed an exponential increase with temperature increment. The effects were so positive that the synthesis and material costs for the silica membranes were more than compensated in comparison to the polymeric counterparts. Selectivity was also found to increase and maintain a high magnitude [64]. Verification for this dependence was performed by the investigation of two alternative mechanisms for their validity in the cases of pure component pervaporation of water, methanol and higher alcohols. The linear trends between the natural logarithm of permeability and temperature inverse suggest the precedence of the adsorption–diffusion model. Based on the exponential relation between flux and temperature, it is predicted that the fluxes for each of the components shall increase with temperature and due to an unequal increment, selectivity will be positively affected. These were supported by experimental results [71]. It was clear that pervaporation based total fluxes display an Arrhenius like temperature dependence thereby indicating the activated nature of this process. Furthermore, when the total fluxes were analyzed for methanol–water and ethanol–water mixtures and plotted against temperature with the response of different compositions, the difference in their sensitivity was observed. The increase in the total flux for low alcohol content aqueous mixtures were more affected than the alcohol rich mixtures. Separation factors for alcohol deficient mixtures-maintained constancy; however, the alcohol rich components showed an increase. The temperature increases also negatively impacted the membrane permeability as evidenced by the existence of negative activation energy [43]. The implementation of the solution-diffusion model to predict the total as well as partial fluxes makes use of an Arrhenius type temperature dependence on temperature. The agreement with experimental results via straight-line plots between natural log of permeation flux and inverse temperature (Figure 9) confirms this model [72].

Temperature based studies are also useful for determining the mechanism responsible for mass transport in the membrane. It was postulated that for membranes having pore sizes that demonstrate viscous flow mechanism, temperature should not have an impact on the product of permeability and liquid viscosity [42]. Temperature dependency of the product of solvent permeability and pore localized solution viscosity indicates a shift from the Hagen–Poiseuille flow regime. Other than affecting the fluxes, this is also an important implication obtained by investigation utilizing temperature variation to act as a window for narrowing down the possible mode of transmembrane mass transport. Alkane flux was unaffected, whereas alcohol rejection was decreased with an increase in temperature for reverse osmosis [88]. The correlation between temperature and flux data were also carried out and compared for different organic species. This indicated a window into understanding on how these correlations are impacted as a function of the properties of the organic molecules. For instance, the total fluxes and selectivities were increased for aqueous mixtures of dioxane, methanol and DMF. Among the three organic components, dioxane showed greater fluxes and selectivities for the range of temperature investigated, followed by methanol then DMF [73].

As the manufacturing techniques evolved and improved to give rise to doped silica membranes as well as other modifications to the pervaporation module, new data emerged to understand whether these changes affected the temperature and flux dependence. In a study, butanol and propanol were subjected to pervaporative dehydration by titania supported microporous silica membranes. According to the adsorption–diffusion model, flux and selectivity were enhanced at the higher end of the temperature spectrum. This can be justified with an improvement in the driving force caused by an increase in each of the feed component’s vapor pressure and a simultaneous improvement in the mobility with respect to the adsorption process [84]. Pervaporation studies on phenyl-functionalized alumina supported silica membranes for aqueous mixtures of ethyl acetate, methyl ethyl ketone and isopropanol showed an increase in the organic component flux with temperature [1]. Hexamethyldisilazane modified silica membranes showed an increase in the total fluxes of both ethanol–water and acetone–water with temperature with the selectivity being constant [73]. Cobalt doped silica membranes displayed an increase in both water and ethanol fluxes of the binary mixture with temperature. The cause for this trend was credited to the strengthening of the driving force as the vapor pressure of the feed had increased due to elevated temperatures [82]. Silica membrane doped with iron and cobalt, operating via the mechanism of molecular sieving also displays an increase in the total permeation flux with temperature. The concentration factor was also found to increase with temperature; however, operations at higher temperatures do not maintain a stable value with the passage of time [103].

High operating temperatures are preferred, which increase the flux exponentially and thereby requiring less membrane areas and hence reducing the material costs. Silica membranes are able to endure and preserve their performance at high temperatures making them suitable for pervaporation-based applications in contrast to the polymeric membranes. The separation factor as well as the partial component and total fluxes are affected positively by an increase in the operating temperature. In addition to this, studies investigating the dependence of various parameters offer a valuable insight into the governing mechanism responsible for the mass transport across the membrane. A comprehensive understanding of the mechanism opens up the possibilities for modifications and fabrication of membranes highly suitable to a particular process.

### 6.2. Effect of Feed Composition

The adoption of silica membrane based pervaporation for large scale industrial operations is only guaranteed if there is constancy in the composition of permeate. Matters are complicated by feed compositions, which are very much varied across effluent streams that are arising from a wide spectrum of industrial applications. The effluent streams can also be subjected to varying feed compositions with respect to time. In this scenario, the versatility of a membrane is ensured when it can accommodate these changes and offer a steady and stable output. It is here where an understanding of the effects of feed composition on the pervaporation performance of a particular module becomes essential.

In one of the earliest studies, the acetic acid–water mixtures showed an increase in the separation factor with an increase in the acid concentration. Propionic acid–water mixtures also showed the same trends. Both water and acid fluxes were deteriorated with increasing acid concentration on the feed side [46]. In another instance, the water flux was found to increase with an increase in the water concentration in the isopropanol–water binary mixture. The alcohol flux showed a non-monotonic growth and displayed a maximum. This indicates towards the reasoning that the water flux corresponds to its own driving force, whereas the alcohol flux results from the additive contributions of its own driving force and that of water’s [60]. An extensive study by Asaeda et al. [2] displayed the trends of water and isopropanol fluxes along with the separation factor against isopropanol concentration in the feed mixture. The membranes were also subjected to four consecutive runs and the same data were captured and compared. For each of the runs, the water flux was reproducible showing no temporal fluctuations, and in all runs, decreased monotonically. The isopropanol flux on the other hand increased and then decreased showing maxima near the vicinity of the 20 mol% IPA. The reason for such non-monotonic behavior can be attributed to the increase in the number of the adsorbed alcohol molecules on the pore surface, thereby hindering the permeation of the alcohol, as the effective pore diameter has been reduced. This is further complicated by the reaction of isopropanol with surface hydroxyl groups [104]. Besides the commonly investigated alcohols such as ethanol and isopropanol, it was found that the selectivity was decreased with increasing feed water concentration and the fluxes for n-butanol, i-butanol and 2-ethylhexanol was increased [80].

The advent of commercial silica membranes led to a spur in investigations centered on their performance. The water flux was observed to increase with feed water concentration for both the membranes manufactured by Sulzer Chemtech (Linden, Germany) and Pervatech BV (Rijssen, The Netherlands) for an industrial ketonic solution. However, the selectivity was decreased [68]. The study by Casado et al. [70] showed that for the commercial silica membrane PVP (supplied by Pervatech, Rijssen, The Netherlands), isopropanol–water mixtures showed an increase in the water flux with an increase in the feed water concentration. The increments were more pronounced as the operating temperature was successively increased. Commercial silica membranes from Pervatech BV (The Netherlands) were tested against the water–n-butanol binary mixture. Increase in the feed water concentration has led to an increase in the total permeation flux for the range of temperature studied [72].

Comparative studies between silica membranes and other alternatives were also carried out. It was found out in a comprehensive study [54] that the silica membrane behaved in a similar manner as was observed for its zeolitic counterparts. Methanol–water, ethanol–water and n-butanol–water mixtures displayed an increase in the flux of each of the components with an increase in the feed water concentration. Similar trends were observed in a separate study [61] for isopropanol–water pervaporation experimental results, which depicted an increase in the fluxes of both components of the binary mixture as the feed water concentration was increased. In another study [84], γ-alumina and α-alumina supported silica membranes were subjected to 2-butanol and 2-propanol water mixtures and the fluxes for both the systems were increased, however, the selectivity decreased as the water feed concentration was increased. The reason for the diminishing separation factor was linked to the drag effect enforced by the interactions of the alcohol molecule with the large water flux. Pervaporation of acetic acid and propionic acid water mixtures [3] showed a decrease in the organic acid and water fluxes with a decrease in the feed water concentration. The separation factor however increased in this instance. The decrease in the acid flux was causated to the preferential adsorption of the acid molecules to the membrane surface. The total mass flux for the methanol–water and ethanol–water mixtures was also found to increase with an increase in the water concentration in the feed. The increments in the flux with this variation were more pronounced as the temperature was also increased. Linear trends were observed for alcohol rich mixtures, which then developed a non-linear trend at dilute concentrations [43].

**Figure 10:**
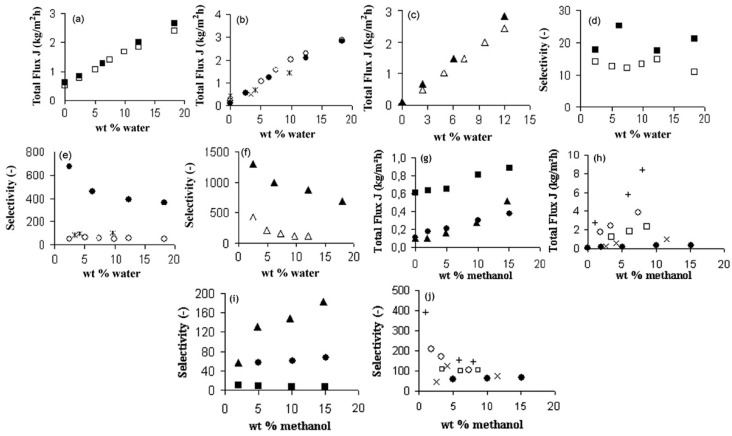
Mass fluxes (**a**–**c**) and separation factors (**d**–**f**) as a function of the water content (wt %) in the feed at 60 °C. Relative error = 3% (**a**–**c**) and 14% (**d**–**f**), (■) M1, ethanol–water, (□) M2, ethanol–water, (•) M1, isopropanol–water, (○) M2, isopropanol–water, (x) M3, isopropanol–water, (▲) M1, butanol–water and (∆) M2, butanol–water. Total mass fluxes (**g**,**h**) and separation factors (**i**,**j**) as a function of the methanol content (wt %) in the feed. Relative error = 3% (**g**,**h**) and 14% (**i**,**j**) (■) M1, ethanol–methanol, 60 °C, (•) M1, isopropanol–methanol, 60 °C, (▲) M1, butanol–methanol, 60 °C, (x) M4, isopropanol–methanol, 60 °C, (□) M4, isopropanol–methanol, 90 °C, (○) M4, isopropanol–methanol, 120 °C and (+)M4, isopropanol–methanol, 150 °C. Adapted from [74], with permission from Copyright (2010), Elsevier Ltd.

The effect of the variation of water and methanol concentration on their respective mixtures with ethanol, isopropanol and butanol were observed. Methylated silica membrane was considered [74]. For the water mixtures of the three alcohols under investigation, the total flux was increased, whereas the selectivity was decreased (Figure 10). The increased total flux occurs due to the rapid increase in the water flux. The selectivity deteriorates due to the interaction between the water and alcohol molecules, widely known as the drag effect. The substitution of water with methanol did not yield any difference in the trends of a total flux for ethanol, isopropanol or butanol; with the flux increasing with the methanol feed concentration regardless of the operating temperature. The separation factor for the ethanol–methanol case decreased as characteristic of the aqueous mixtures however, it increased with the methanol feed concentration for the isopropanol and butanol cases. It was also found that the deviation from linear dependence of the flux with its own driving force initiates sooner for the water based systems than the methanol based systems as the concentration of the either upstream water or methanol is increased in their respective cases. The competition between methanol and the higher homologue for the adsorption sites hinders the flux of the latter. There are two competing interactions at play, the competitive adsorption and drag effect. Small pore membranes, competitive adsorption wins, and the alcohol flux decreased when the water concentration increased on the feed side. Large pore membranes drag effect predominates, and alcohol flux increased with increasing water concentration.

The effect of upstream ethyl acetate concentration increased both the ethyl acetate and water flux however this has led to a decrease in the selectivity for a hydrophobic phenyl functionalized silica membrane [1]. Hexamethyldisilazane surface-modified mesoporous silica membrane when tested for the pervaporative separation of ethanol–water and acetone–water mixtures, the total flux was increased with the upstream organic component concentration accompanied by a decrease in the separation factor [75]. The increase in feed ethanol concentration brought about a decrease in water and ethanol flux, but an increase in the selectivity for cobalt-doped silica membranes [76].

### 6.3. Effect of Transmembrane Pressure Difference

The applied pressure is one of the user dependent input features that cause pervaporation based separations as well as dehydrations in the first place. Variation of this parameter has a large impact on the performance related parameters such as the separation factor or selectivity as well as total and partial fluxes. Theoretical modeling of the phenomenon was postulated towards the dependence of flux on a driving force corresponding to each component in the feed mixture. By studying the effects of variation of the applied pressure, useful insights can be gathered in better understanding of these driving forces and their interactions with the permeating species. Preliminary studies were focused on the magnitudes of the applied pressure, however with the advent of better implementations of various models, the focus shifted to transmembrane partial pressure differences. This was done because these differences constitute the driving force for each component and are not limited to affecting the flux of its corresponding permeating species.

In a comprehensive study, it was observed for the three silica-zirconia composite membranes of having pore diameters of 2.9, 1.6 and 1.0 nm that the permeate volume fluxes for methanol, ethanol, isopropanol and butanol increase with the magnitude of the applied pressure [56]. Expressions for the flux for each of the species in the mixture under investigation were derived from the Maxwell–Stefan model. The water flux was found to be proportional to the difference between its partial equilibrium vapor pressure of the retentate and permeate side. The alcohol flux was proportional to the difference of its partial equilibrium vapor pressure between the retentate and permeate side along with an additional term addressing the drag effect from the water molecules. An increase in the permeate pressure decreased the magnitude of this driving force for the water flux. Since the alcohol flux is related with the water flux, it also decreased with this increase. The separation factor decreases with permeate pressure [60]. In a separate study [66], the water flux was found to be proportional to its driving force, i.e., the partial pressure difference. It was also found to be the highest for ketone– and ether–water systems. The viscosity also played an important role and the flux was observed to decrease with an increase in the viscosity. The water fluxes in the water–DMF, water–methanol and water–dioxane mixtures showed a near linear trend suggesting proportionality with the partial pressure difference across the membrane. The absolute values of the fluxes, however, were subject to the pore blocking effects by the accompanying organic component [73]. In another instance [69], the pervaporation across various constituents comprising of the supported silica membrane module were inspected with respect to the transmembrane pressure gradient. Since the pore sizes in the α-alumina layer are characteristic of the viscous flow model, the flux through it is directly proportional to the pressure gradient across the membrane. γ-alumina membranes exhibited a threshold pressure for some hydrophobic solvents, whereas this was not the case for hydrophilic solvents, which had stronger affinity for the membrane. MCM-48 has the smallest pore sizes and it is here where the nature of the interactions between the solvent and membrane become more dominant. The flux can be affected by the alkoxylation of the surface silanol groups by the alcohol molecules and thereby reducing the effective pore size. For the ethanol–water (90:10 wt %) [63], the water flux in the case of the zeolite membrane showed a linear dependence with the transmembrane partial pressure difference, or the driving force and displaying near constancy in the selectivity. Water flux and selectivity were decreased with a decrease in the driving force for the silica membrane. A microporous silica membrane supported by γ-alumina and α-alumina layers was subjected to pervaporation for 2-butanol and 2-propanol water mixtures [84]. Maxwell–Stefan modeling was chosen and the experimental results showed the increment in the water flux with its partial pressure difference, whereas when the alcohols were investigated for their fluxes against their own driving force, they were marked by a decrease and hence showed the predominant coupling effect of the water flux on their permeation. The permeate weight flux for decanol–ethanol and decane–ethanol showed a linear increase with respect to the applied pressure and an increase with increased temperature due to the reduced viscosity [88]. The effect of increasing the permeate pressure implies an increase in the partial pressure of the permeating component and hence a decrease in the magnitude of the difference of partial pressure across the membrane. This leads to a decrease in the driving force and thereby affecting the flux. The total permeation flux for the n-butanol–water mixture across the commercial Pervatech BV membrane corroborated the theoretical predictions. The separation factor was found to decrease marginally [72].

**Figure 11:**
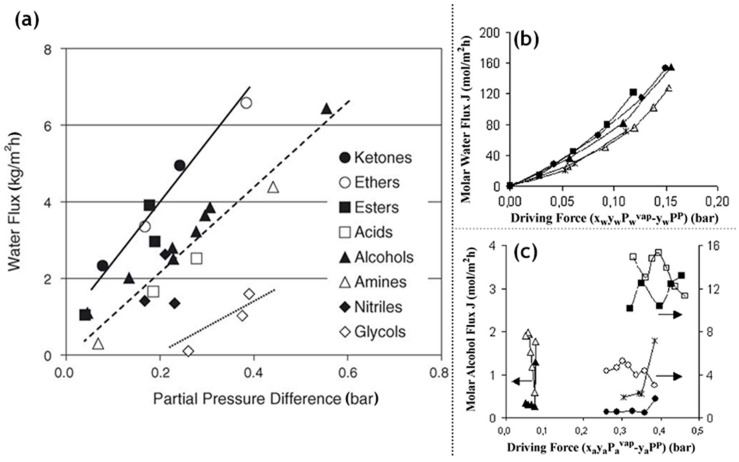
(**a**) Partial permeate flux of water over partial pressure difference for the ECN silica membrane with different systems in pervaporation at a feed concentration of 10 wt % water, a permeate vacuum of 20 mbar and feed temperatures below the boiling point at ambient pressure. Lines are added as a guide to the eye. Adapted from [65], with permission from Copyright (2005), Elsevier B.V. (**b**) Molar water flux as a function of the water driving force at 60 °C. Relative error = 6%. (**c**) Molar alcohol flux as a function of the alcohol driving force for aqueous mixtures at 60 °C. Relative error = 6%: (■) M1, ethanol–water, (•) M1, isopropanol–water, (▲) M1, butanol–water, (∆) M2, butanol–water, (x) M3, isopropanol–water and (□) M2, ethanol–water. The full lines are a guide to the eye. Adapted from [74], with permission from Copyright (2010), Elsevier Ltd.

Some interesting observations were obtained in the cases of the plots for methanol–water and ethanol–water binary mixtures [43]. The flux for each component was plotted as a function of their driving force for each of the two systems. The flux increased for water in methanol and increased gradually but then increased steeply for pressure differences around 0.2 bar. Methanol showed a linear increase of its flux in response to an increase in its driving force showing independence from the driver force for water. Fick’s law of binary diffusion can be applied here. On the other hand, ethanol showed a non-linear decrease in its flux with an increase in its driving force (Figure 11). The adsorption on the membrane is not as strong as methanol and hence it experiences a drag effect from the water molecules and the Fick’s description can no longer encapsulate this flow. From the work of Asaeda et al. [2], it was concluded that the water flux was subject to influence from the upstream partial water vapor, but it was not directly proportional to the partial equilibrium vapor pressure difference. Further, corroboration ensued when a non-linear dependence between the water flux and its driving force was observed (Figure 11) for ethanol–, isopropanol– and butanol–water mixtures [74]. For the same driving force, the highest water flux occurred when it was present with the alcohol of having lowest molecular mass. This can also be due to a higher capability of butanol to block the pores than ethanol. The plots of alcohol flux against their driving force displayed a strong hindrance to their permeation by water. For alcohol–methanol systems, methanol flux was directly proportional to its driving force, however the linearity is not observed for high methanol concentration systems. The isopropanol flux increased with an increase in its driving force for lower temperatures, but it displayed the opposite behavior at higher temperatures.

There is another discernable feature that promotes interpretation from the trend of water flux with respect to the partial pressure difference. The slope of these straight-line plots gives a measure of the permeance of the water when coupled with an organic component [65]. From the plethora of organic compound–water mixtures studied for their pervaporation performance against the commercial ECN silica membranes, highest fluxes were characteristic of ketones, ethers and esters, whereas glycols ranked the lowest. Satisfactory fluxes were obtained for the rest spanning the alcohols, amines, nitriles and the acids. In addition to the validity of the integral transport equation derived from the Maxwell–Stefan model, the competitiveness for the adsorption sites and the viscosity of the water–organic component systems can be judged.

### 6.4. Membrane Stability

The advantages of the silica membranes are well known, encompassing mechanical strength, thermal stability and resistance to swelling in organic–water mixtures. However, their susceptibility to failure when exposed to aqueous or dilute feed compositions due to their inherent hydrophilic character severely impedes their pervaporation performance. In this regard, numerous studies have also dedicated a separate section for assessing the long-term performance of the synthesized or commercial membranes rather than only reporting the performance related metric of just synthesized or obtained silica membranes. In this regard, studies focusing on the stability of membranes have been collected and presented to better acquaint the reader with the method of ascertaining the stability of a particular membrane for pervaporation-based applications. The stability of the membrane also has a direct bearing on the economic feasibility of the pervaporation process for an application. These have been quantified to a large extent based on practical observations of such units. Membranes replacement costs have been calculated; however, these need an update as the methods of manufacturing have drastically altered [63].

**Figure 12:**
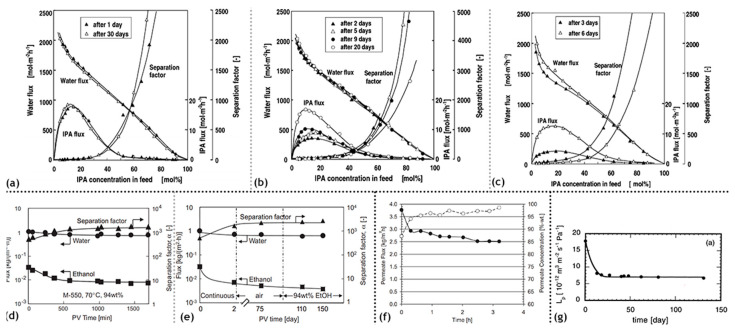
(**a**) Observed pervaporation characteristics for isopropanol/water mixtures at normal boiling points with a SiO_2_–ZrO_2_ (ZrO_2_: 40%) membrane fired at 400 °C (with washing in boiling water). (**b**) Observed pervaporation characteristics for IPA/water mixtures at normal boiling points with a SiO_2_–ZrO_2_ (ZrO_2_: 30%) membrane fired at 400 °C (with washing in boiling water). (**c**) Observed pervaporation characteristics for isopropanol/water mixtures at normal boiling points with a SiO_2_–ZrO_2_ (ZrO_2_: 20%) membrane fired at 400 °C (with washing in boiling water). Adapted from [2], with permission from Copyright (2002), Elsevier Science B.V. (**d**) Time course of pervaporation performance using a fresh Co–SiO_2_ membrane M-550 (ethanol feed concentration of 94 wt %, 70 °C). (**e**) Long-term PV performance of Co–SiO_2_ membrane M-550 (top layer fired at 550 °C, dry air). After about 2 days of continuous PV, the membrane was kept in air and then was immersed in 94 wt % ethanol for a total of 150 days. The initial PV performances (fresh membrane) are shown at a PV time of 0. Adapted from [76], with permission from Copyright (2010), Elsevier B.V. (**f**) Permeate flux and purity over operation time for the system IPA/water at 5 wt % in the feed and 80 °C in pervaporation showing the conditioning effect of the silica membranes. Open symbols and dashed line are for permeate concentration. Filled symbols and the solid line are for the permeate flux. Adapted from [64], with permission from Copyright (2005), Elsevier Ltd. (**g**) Time course of pure water permeabilities (@P = 1.0 MPa, pH = 5, 25 °C). Adapted from [56], with permission from Copyright (1998), Elsevier Science B.V.

There was a prolonged interest in doping silica membranes with other oxides such as zirconia and titania to affect any changes in the stability of otherwise unaltered silica membranes. In a study [56], silica-zirconia composite membranes synthesized for the purpose of nanofiltration were immersed in the test solution and the resultant flux data were recorded at regular intervals of two days for a hundred-day period. A transmembrane pressure of 1 MPa was employed and the results indicated constancy in the flux data after some measurable decrease in the first one-two days. The addition of zirconia improved the long-standing drawback of water induced susceptibility of the silica membranes. In another study [2], silica-zirconia membranes were inspected for their structural homogeneity. The membranes were subjected to multiple runs against various concentrations of isopropanol–water mixtures. The conditions were made more taxing on the membranes by using aqueous solutions of isopropanol for time durations ranging from 12 h to months (Figure 12). The first run displayed a constant water flux while the alcohol flux decreased dramatically to yield high separation factor in a matter of few hours. The reason for this decrease has been attributed to the adsorption of isopropanol molecules on to the membrane surface and thereby reducing the effective diameter. Subsequent runs were evenly spaced out for 12 h duration while being kept immersed in a dilute aqueous isopropanol solution. X-Ray diffraction patterns displayed the existence of discrete zirconia phases, thereby implying the presence of silica phases. Low temperature fired membranes displayed favorably large water fluxes and an impressive stability of up to 2.5 months. Silica membranes and its doped variants were studied for their chemical stabilities [81]. Alumina doping led to deterioration in the chemical stability, whereas doping with zirconia and titania improved the resistance to high pH solutions. In the case of normal silica membranes, it can tolerate a pH range of 2–8, 10 mol% zirconia and titania improved the range to pH of 2–10. Doping levels of 30 mol% however destroyed the structural integrity of the membrane. The inherent affinity of a material to be soluble towards a particular component comprising of the feed mixture plays an important role in deciding its ability to affect pervaporation-based separations [3]. Silica-zirconia membranes failed after a few hours when operating in an acetic acid–water mixture. On the other hand, silica-titania membranes were very stable, but yielded very low flux values. These results were reminiscent of their solubility against aqueous acetic acid mixtures. The subsequent results signaled towards the versatility of the silica membrane devoid of any apparent doping. This was because they provided a balance between these two opposite extremes. Cobalt-doped sol–gel synthesized silica membranes were investigated for their flux values for a period encompassing 150 days [76]. An initial decrease in the ethanol and water flux was recorded and the separation factor was found to increase. However, after a brief period of change, the resultant values stabilized.

Pervaporation of the commercial silica membrane prepared by ECN showed remarkable performance and reproducible results at high temperatures [59]. The temperature range achieved by this membrane overshadows the operating range required by the polymeric membranes. Single step and two step hydrolyzed methylated silica membranes were tested against their stability at different operating temperatures [15]. Pervaporation at 95 °C displayed a marked decrease in the water flux, which might be due to the adsorption of the water molecules in the membrane surface. The increase in the methylation causes more hydrophobicity thereby reducing the water flux. Pervaporative dehydrations at 115, 135 and 165 °C caused membrane failures within a span of 4–40 days. Reorganization of the membrane at the molecular level may be the cause of the formations of cracks or pinholes as indicated by various scanning electron micrographs. The required membrane stability for long term operations can be determined by prescribed process conditions requiring only two weeks [55]. Good reproducibility of the permeate fluxes is essential for ensuring uniformity, a predisposed condition for industrial adoption of the process. Amorphous silica membranes were operated for a period of 73 days for an industrial effluent stream comprising of water, isopropanol and other impurities such as heavy hydrocarbons, wherein polymeric membranes are useless in such a situation. An operating temperature of 80 °C and 25 mbar permeate pressure showed a decrease in both isopropanol and water flux however, in such a manner that the selectivity was increased. This change may be attributed to the change in the surface silanol groups. Long time operations displaying constancy in results can lead to the acceptance or rejection for the large-scale industrial uptake of this process against the conventional practices in place. The hybrid organic–inorganic membrane synthesized by the collaboration between Universities of Twente and Amsterdam and the WEnergy Research Center of the Netherlands displays remarkable hydrothermal stability, constant and continuous operation exceeding a year at semi-industrial process conditions [78].

## 7. Conclusions

The superiority of inorganic silica based membranes can be ascertained beyond doubt over the polymer based organic membranes. It has been shown in numerous studies and pervaporation setups that this precedence stems out primarily from where organic membranes begin to degrade. They cannot operate at higher temperatures in comparison to silica membranes or their modified variety. High temperature operations are desirable as this process condition has a constructive impact on both selectivity and flux. This is particularly important as it is observed that almost membranes in one form or the other are bound by the trade-off between these two performance parameters. Increment in flux is always observed at the cost of reduced selectivity and vice-versa. However, as observed in almost all pervaporation studies on membranes, higher operating temperatures tend to shift this trade-off towards higher more practical and industrially viable magnitudes. The deterioration or the inherent instability possessed by polymer based organic membranes can only be overcome by their substitution with silica based inorganic membranes.

Silica membranes require a support that can be constituted of either α-alumina or γ-alumina or both in conjunction. Early studies into the synthesis of silica membranes for the purpose of pervaporation utilized the α-alumina support layer. This support layer has a larger pore size than the γ-alumina layer and hence permits higher fluxes as well as reduced flow resistance. However, it comes at a cost of associated structural imperfections as the difference between the α-alumina pore size and the typical pore size encountered in silica membranes is large, differing by an order of magnitude. This difference leads to the penetration of the silica sol into the support layer during the dip-coating process. In addition to this due to less contact between the silica top layer and the α-alumina support layer, these membrane modules are more susceptible to developing cracks and/or pinholes, which are imperfections that substantially reduce the selectivity of a pervaporation module, especially when molecular sieving regime is desired. γ-alumina layers have pore sizes in the intermediate range. The replacement of α-alumina with γ-alumina can overcome these structural disadvantages. However, the complete replacement of α-alumina layer is impractical as it will lead to higher thickness of the γ-alumina layer thereby resulting in much higher flow resistance and dismal fluxes of little practical significance. Hence, a compromise of these two support layer materials is advisable. The silica top layer is highly selective, and hence extremely thin layers are desirable. This is followed by the γ-alumina interlayer, which serves to support the silica top layer and prevent cracks or pinholes to form during or after operation. A suitable thickness of this material is desirable, setting a compromise between flow resistance and structural integrity. Finally, α-alumina base layer succeeds these two and serves as the backbone of the module, providing support to the other layers that are most likely to degrade if operated with the same thickness but in the absence of the α-alumina base layer. Therefore, a module with a successive silica, γ- and α-alumina layer is the most desirable as it can handle high transmembrane pressure difference, thermal loads as well as chemical resistance against a variety of solvents.

Although a circular disk like the pervaporation module maybe of more importance from the academic point of view, tubular modules are favored for the purpose of large-scale industrial applications. Tubular modules make use of the dip-coating route of sol deposition in contrast to the slip-casting route employed for the synthesis of the former. The dip-coating process is simpler and does not require high rpm rotations and can be achieved by simple dipping of the tubular support layers in the silica sol. Gelation is achieved as the sol retreats from the surface and a thin layer is achieved. There does exist a problem of sol accumulation near the edges, however, this can be made more uniform by quicker repetitive dipping of the module into the sol thus ensuring greater uniformity of the thickness of the selective silica top layer. Numerous studies almost always use manufactured α- and γ-alumina support layers and most studies differ in the subsequent steps of either using commercial fully manufactured modules or modifying the silica sols for achieving modifications and properties, unobtainable using commercial alternatives. Many commercial modules have been highlighted in the study. Their repetitive use against a variety of solvents in water deficient mixtures has been studied extensively by numerous research groups. This can be attributed greatly to their success of being chemically resistance for a large pH range. Due to the uniformity in the membrane parameters, attributed to being produced by the same manufacturer, these membranes have yielded a deeper insight into understanding the governing mechanism responsible for transmembrane mass transport. This laid the empirical groundwork for establishing relations between structural properties, operating conditions and the resultant pervaporation performance parameters. However, they could not be modified further, and hence silica sols prepared in-house gave the freedom to come up with modifications that sought to overcome the disadvantages of silica based inorganic membranes. Methylated as well as phenyl functionalized silica membranes could handle feed compositions that were water rich without perceptible changes in the results for an extended period. This was due to the enhanced hydrophobic nature in contrast to the hydrophilic nature of silica membranes owing to the possibility of forming hydrogen bonds between the surface silanol groups and water molecules.

It can be gleaned from the extensive studies focused on achieving pervaporation using silica membranes that due to the absence of a definitive and optimal way of manufacturing these types of membranes, earlier investigations were focused only on the synthesis of the silica membranes. As the solution–gelation route of manufacturing pervaporation friendly membranes gained acceptance, it led to the realization of few commercial silica membranes. These proved to be highly successful based on the numerous studies that followed, which sought to test these membranes against a large matrix of feed compositions varying in both the concentrations as well as the organic component itself. There were studies focused also on comparing and setting up a precedence order among these commercial silica membrane varieties as well as between their polymeric and zeolitic counterparts. It was concluded that the zeolite membranes were superior in certain aspects whereas silica membranes offered good flux however moderate selectivity. Silica membranes still were more suitable due to their easy disposability, manufacturability and resilience. The existence of a certain standard in the form of a commercial silica membrane, against which different feed conditions could be tested along with the interplay of operating temperature and transmembrane pressures spurned out the validity of various models that sought to describe the transmembrane mass transport. The adsorption–diffusion model proposing the Arrhenius type dependence of flux on temperature gained precedence. However, it was being realized that the commercial silica membranes were not entirely impervious to faults and hence their associated hydrophilic nature came into scrutiny. It was found that these membranes could handle water deficient organic mixtures but failed against water rich feed compositions thereby, the time dependent performance of a membrane began to be addressed as a separate and important aspect known as stability. Studies focused on in-house manufactured silica sols or subsequent modifications to the commercial silica membranes gained traction. These modified membranes, which had the objective of reducing or eliminating the hydrophilic interactions between surface silanol groups and water molecules were found to be stable for extended periods of time yielding reproducible results.

A membrane suited for industrial pervaporation applications should possess the ability to operate at high temperatures, high transmembrane pressures, possess high chemical resistance and yield reproducible results for an extended period. It was observed by Sommer et al. [66] that the complete substitution of the distillation process with the membrane-based pervaporation process was not feasible. Conventional distillation performed better against feed compositions that were skewed concentration wise towards either of the component in the binary mixture but performed poorly against compositions approaching azeotropes. The membrane based pervaporation technique for separation expended a large amount of time for separating organic solvent rich or poor feed compositions however, reigned superior when dealing with azeotropes or close to azeotropic compositions mixtures. The process setup that sought to implement both separation techniques required lesser energy, achieved a higher degree of separation and resulted in lower maintenance and operating costs. In addition to this, a pervaporation module should possess high heat and mass transfer coefficients, uniform flow distribution, ease of replacement and maintenance and a low manufacturing cost. Turbulent flow regime is favorable to overcome temperature and concentration polarizations near the surface of the module [105].

Mesoporous silica membranes are poised to dominate separation technologies as they offer a potential for establishing circular economy, convenient waste disposal and lower operating as well as maintenance costs in contrast to distillation and other conventional technologies. It is evident from the extensive investigations conducted by numerous research groups that these inorganic silica-based pervaporation friendly membranes offer operations at higher temperature, more thermally, chemically and mechanically resilient alternative and higher reproducibility of the permeate composition in comparison to the polymeric and zeolitic membranes in existence. There still exists an enormous potential in synthesizing more customized membranes serving optimum performance with regards to the feed composition, temperature as well as transmembrane pressure as the governing mechanism for mass transport requires further understanding to overcome the limitations of approximate models. Membrane stability with respect to prolonged exposure to extreme feed conditions requires further investigations and hence inevitably remains the least pondered aspect of these versatile membranes. The issue of the inherent hydrophilic character of silica has been addressed; however, many studies still focus on organic component rich feed compositions thereby yielding favorable results that need to be avoided. Finally, the ever-growing mature field of silica membrane synthesis for pervaporation applications remains viable to undergo further disruptive innovations and the point of saturation is still far ahead in the future.

## Figures and Tables

**Table 1:** Specifications of silica membranes fabricated in some of the early studies aimed for pervaporation based applications.

Research Group	Pore Size	Thickness
Klein et al. [6]	0.025 mm	150 µm
Kaiser et al. [49]	<2 nm	5–10 µm
Kaiser et al. [50]	0.1 µm	0.5 mm
Attard et al. [51]	≈3 nm	-
Brinker et al. [52]	0–3.1 nm	-
Gallagher et al. [53]	0.005–0.065 nm	0.1 mm
Ogawa et al. [54]	1.8 nm	1.5 nm
Yazawa et al. [55]	<2 nm	2 µm
Kitao et al. [46]	<0.5 nm	10 µm
Brinker et al. [47]	<1 nm	20–120 nm
Ryoo et al. [48]	1.6 nm	0.5 mm
Tsuru et al. [49]	1.0–2.9 nm	-
Roh et al. [50]	1.6 nm	1.9 nm

**Table 2:** Pervaporation performance for various solvents in their aqueous solutions, separation achieved using the commercial silica membrane, ECN (Petten, The Netherlands). Adapted from [59,60] with permission from Copyright (2001), Elsevier Science B.V.

Organic Component	Water Concentration (wt%)	Temperature (°C)	Permeate Pressure (mbar)	Separation Factor	Water Flux (g/m^2^h)	Reference
Ethanol	3.6	70	6	350	1485	Veen et al. [59]
Ethanol	4.5	71	5	208	1220	
IPA	4.5	80	25	1150	1855	
n-BuOH	5	75	10	600	4500	
1,2-dichloroethane	0.24	70	10	39,645	964	
triethyleneglycol	9	80	8	2054	184	
ethylenedimine	30	75	10	210	28	
acetonitrile	10	70	10	100	2630	
MEK	2.5	66	10	1458	2280	
acetone	10	50	6	33	752	
EA	2	70	8	1118	2936	
DMF	5	75	10	24	189	
DMF	5	100	10	102	1007	
THF	5	60	11	147	5819	
isopropanol	5	70	10	600	2100	Verkerk et al. [60]
n-BuOH	5	70	10	680	2300	

**Table 3:** Pervaporation performance for various solvents in their aqueous solutions, separation achieved using the commercial silica membrane, ECN (The Netherlands). Adapted from [71], with permission from Copyright (2005), Elsevier Science B.V.

Organic Component	Water Concentration (wt%)	Temperature (°C)	Permeate Pressure (mbar)	Separation Factor	Water Flux (g/m^2^h)
methanol	10.4	60	11	10	1870
ethanol	10.3	70	14	60	2330
IPA	10.2	75	12	90	2760
n-BuOH	8.6	75	10	930	3690
*sec-BuOH*	10.2	75	8	850	3250
*iso-BuOH*	9.6	75	9	1200	3880
*tert-BuOH*	7.2	75	9	1100	2830
Cyclohexanol	12.5	90	10	1200	3210
Ethylene glycol	10.5	100	8	880	100
1,2-Propanediol	9	100	8	3400	1030
1,4-Butandiol	8.8	100	8	2600	1600
Phenol	4.7	100	13	4500	6460
Acetone	11.5	50	12	70	2630
MEK	7.6	70	12	300	5150
MIBK	1.4	85	8	9400	1910
Formic acid	11.6	80	16	2	17,480
Acetic acid	10.4	80	12	60	1910
Propionic acid	8.4	80	9	250	2630
Ethyl acetate	2	70	24	750	3160
*n -Butyl acetate*	0.5	75	20	160,000	1050
Ethyl lactate	9.8	70	22	230	4060
1,4-Dioxane	7.9	84	11	520	6730
THF	11.8	60	12	210	3470
Acetonitrile	11.9	70	10	200	2730
DMAc	13.5	80	12	180	1600
DMF	10.2	80	12	100	1530
Diethylamine	11	49	8	8100	300
Triethylamine	3.9	80	20	650	4550
Ethylenediamine	10	100	8	500	30
Dichloromethane	0.3	37	8	2100	540
Chloroform	0.1	50	7	4700	510
1,2-Dichloroethane	0.6	70	8	150,000	1080
Trichloroethylene	0.1	70	8	3500	240
Tetrachloroethylene	0.04	100	8	3400	450

**Table 4:** Pervaporation performance for various solvents in their aqueous solutions, separation achieved using the commercial silica membrane, Pervap SMS.

Organic Component	Water Concentration (wt%)	Temperature (°C)	Permeate Pressure (mbar)	Separation Factor	Water Flux (g/m^2^h)	Reference
t-BuOH	15.7	60	8–10	175	350	Gallego-Lizon et al. [67]
	10.6	60	8–10	144	350	
	5.7	60	8–10	142	350	
	18.7	80	8–10	168	850	
	15.2	80	8–10	427	850	
	9.3	80	8–10	897	850	
	5.8	80	8–10	1268	850	
	2.2	80	8–10	1554	850	
	15.3	100	8–10	253	1200	
	10.7	100	8–10	406	1200	
	5.6	100	8–10	562	1200	
	1.8	100	8–10	540	1200	
	0.3	100	8–10	262	1200	
acetone	0–30	40–70	<8	<14,000	500–1100	Urtiaga et al. [68]
	10	40	<8		380	Casado et al. [70]
	10	70	<8		520	
IPA	5	80	20		1400	Sommer et al. [66]

**Table 5:** Pervaporation performance for various solvents in their aqueous solutions, separation achieved using the commercial silica membrane, PVP (Pervatech BV).

Organic Component	Water Concentration (wt%)	Temperature (°C)	Permeate Pressure (mbar)	Separation Factor	Water Flux (g/m^2^h)	Reference
acetone	20	40–70	<8	<13,000	500–7000	Urtiaga et al. [68]
methanol	5	80	8–10	20	1850 *	Johan et al. [73]
*N*,*N*-dimethylformamide	5	80		130	610 *	
1,4-dioxane	5–22	30–80		120–185	1300–2500 *	
acetone	10	40	<8	1800	440	Casado et al. [70]
	10	70		1800	2720	
IPA	10	40			1300	
	5	70			2000	
	10	70			3200	
	10	90			8200	
Methanol	10.5	60	13	20	3900	Sommer et al. [65]
Ethanol	11	70	12	160	2000	
Isopropylalcohol	9.8	75	13	190	2550	
*n-Butanol*	9.4	75	16	340	4140	
Cyclohexanol	11.2	91	13	4300	2270	
Ethylene glycol	9.7	100	9	67,000	320	
1,2-Propanediol	8.1	100	11	25,000	1540	
1,4-Butandiol	7.5	100	10	120,000	570	
Phenol	6.2	100	18	2800	5070	
Acetone	12.1	50	15	20	3640	
MEK	11.4	60	21	4100	5240	
MIBK	0.8	85	11	29,000	1740	
Ethyl lactate	18.2	70	15	640	4510	
1,4-Dioxane	9.1	85	14	2400	3840	
THF	11.5	60	15	8400	3300	
Acetonitrile	9.7	70	17	330	3900	
DMAc	9.4	80	11	290	2210	
DMF	9.1	80	10	120	1140	
Triethylamine	5.5	70	11	84,000	1350	

* Total flux.

**Table 6:** Pervaporation performance of modified or doped silica membranes for various water–organic mixtures.

Membrane	Composition	Component 2 (wt%)	Temperature (°C)	Permeate Pressure (mbar)	Separation Factor	Water Flux	Reference
methylated SiO_2_	n-BuOH:water	5	95		500–20,000	4000	Campaniello et al. [15]
inhouse(SiO_2_-Ti/α-alumina)	acetic acid:water	10	100		2050	2160	Asaeda et al. [3]
inhouse(SiO_2_-Zr/α-alumina)	THF:water	10	50		3800	7200	
ECN methylated SiO_2_membrane	ethanol:water	6.3	60	<10	25	1280 *	Bettens et al. [74]
		7.5			12	1390 *	
	IPA:water	6.3			460	1220	
		7.5			52	1540	
	n-BuOH:water	6.1			1000	1500	
		7.3			154	1500	
phenyl functionalized SiO_2_/γ-alumina/α-alumina	ethyl acetate:water	95	39.85	0.4	216 **	200	Araki et al. [1]
		90			65 **	340	
	MEK:water	95			44 **	270	
		90			42 **	190	
	IPA:water	95			20 **	280	
		90			9 **	340	
hexamethyldisilazane modified SiO_2_/asymmetric alumina (NGK Insulators Ltd., Aichi, Japan)	ethanol:aceticacid:water	5:1:94	29.85	<1	8.4	920	Jin et al. [75]
			39.85		8.2	1240	
	acetone:aceticacid:water	5:1:94	29.85		26	1200	
			39.85		24	1850	
	ethanol:water	95	29.85		8.7	920	
Co doped SiO_2_/α-alumina				<10			Wang et al. [76]
(fired at 350 °C)	ethanol:water	5.7	70		65	1200	
(fired at 450 °C)	ethanol:water	5.8	70		346	788	
(fired at 550 °C)	ethanol:water	5.9	70		1675	753	
methylated SiO_2_	ethanol:water	18	60	10	22	2600	Pereira et al. [77]

*—total flux and **—separation factor in terms of the organic component.

**Table 7:** In-house manufactured silica membranes and their pervaporation performance for various water–organic as well as organic–organic mixtures.

Membrane	Composition	Component 2 (wt %)	Temperature	Pressure	Selectivity	Flux	Reference
inhouse (silica/γ-alumina)	methanol:water	2–10	60	2	10–200	60–200 *	van Gemert et al. [79]
	ethanol:water	2–9	70		50–160	150–350 *	
	IPA:water	2–9	70		300–550	200–700 *	
	methanol:methyl tert-butyl ether	94	35		2	59.8 *	
	methanol:methyl tert-butyl ether	91	35		18.7	40.8 *	
	methanol:methyl tert-butyl ether	84.5	35		5.9	37.5 *	
	methanol:methyl tert-butyl ether	93.4	35		3.8	12.7 *	
inhouse	BuOH:water	5	75	<2	250	3000	Cuperus et al. [80]
inhouse	2-BuOH:water	5	80		360	760	Sekulic et al. [81]
inhouse (silica/γ-alumina)	n-BuOH:water	5	80	10	900–1200	1310–2920	Peters et al. [82]
	DMF:water	5	73		17–25	700–360	
inhouse (silica/α-alumina)	acetic acid:water	10	90		280	2250	Kitao et al. [46]
		10	90		400	1450	
inhouse (silica/α-alumina)	acetic acid:water	10	75		800	3050	Asaeda et al. [83]
inhouse (silica/α-alumina)	acetic acid:water	10	75	<5.3	800	3060	Asaeda et al. [3]
		10	100		525	5900	
microporous silica membrane	ethanol:water	7.68	71	20	400	2590	Pereira et al. [77]
		27.5	70		302	5980	
	ethyl lactate:water	12.95	70		590	8150	

* total flux.
